# Body mass index and adiposity influence responses to immune checkpoint inhibition in endometrial cancer

**DOI:** 10.1172/JCI180516

**Published:** 2024-06-20

**Authors:** Nicolás Gómez-Banoy, Eduardo J. Ortiz, Caroline S. Jiang, Christian Dagher, Carlo Sevilla, Jeffrey Girshman, Andrew M. Pagano, Andrew J. Plodkowski, William A. Zammarrelli, Jennifer J. Mueller, Carol Aghajanian, Britta Weigelt, Vicky Makker, Paul Cohen, Juan C. Osorio

**Affiliations:** 1 Laboratory of Molecular Metabolism, The Rockefeller University, New York, New York, USA.; 2 Division of Endocrinology, Department of Medicine, Memorial Sloan Kettering Cancer Center (MSK), New York, New York, USA.; 3 Division of Endocrinology, Diabetes and Metabolism, Department of Medicine, Weill Cornell Medicine, New York, New York, USA.; 4 Department of Radiology, MSK, New York, New York, USA.; 5 Center for Clinical and Translational Science, The Rockefeller University, New York, New York, USA.; 6 Department of Surgery, Gynecology Service,; 7 Department of Medicine, and; 8 Department of Pathology and Laboratory Medicine, MSK, New York, New York, USA.; 9 Laboratory of Molecular Genetics and Immunology, The Rockefeller University, New York, New York, USA.

**Keywords:** Metabolism, Oncology, Adipose tissue, Cancer immunotherapy, Obesity

## Abstract

**BACKGROUND:**

Obesity is the foremost risk factor in the development of endometrial cancer (EC). However, the impact of obesity on the response to immune checkpoint inhibitors (ICI) in EC remains poorly understood. This retrospective study investigates the association among BMI, body fat distribution, and clinical and molecular characteristics of EC patients treated with ICI.

**METHODS:**

We analyzed progression-free survival (PFS) and overall survival (OS) in EC patients treated with ICI, categorized by BMI, fat-mass distribution, and molecular subtypes. Incidence of immune-related adverse events (irAEs) after ICI was also assessed based on BMI status.

**RESULTS:**

524 EC patients were included in the study. Overweight and obese patients exhibited a significantly prolonged PFS and OS compared with normal BMI patients after treatment with ICI. Multivariable Cox’s regression analysis confirmed the independent association of overweight and obesity with improved PFS and OS. Elevated visceral adipose tissue (VAT) was identified as a strong independent predictor for improved PFS to ICI. Associations between obesity and OS/PFS were particularly significant in the copy number–high/*TP53*abnormal (CN-H/*TP53*abn) EC molecular subtype. Finally, obese patients demonstrated a higher irAE rate compared with normal BMI individuals.

**CONCLUSION:**

Obesity is associated with improved outcomes to ICI in EC patients and a higher rate of irAEs. This association is more pronounced in the CN-H/*TP53*abn EC molecular subtype.

**FUNDING:**

NIH/NCI Cancer Center; MSK Gerstner Physician Scholars Program; National Center for Advancing Translational Sciences (NCATS); Cycle for Survival; Breast Cancer Research Foundation.

## Abstract 2

Obesity is a risk factor for developing endometrial cancer; however, it may play a protective role in women with this malignacy being treated with immunotherapy.

## Introduction

Endometrial cancer (EC) constitutes the leading cause of gynecologic cancer–related death in the United States and one of the few cancer types with increasing incidence and disease-associated mortality (1). Obesity is one of the main drivers in the development of EC (2, 3), with a clear stepwise correlation between BMI and the risk of developing EC (4). Elevated body weight is also associated with worse prognosis in patients with this malignancy (5). Mechanistically, obesity induces dysfunction in the adipose tissue (AT), which has been implicated in promoting the progression and growth of EC cells (6) and triggering a dysregulated inflammatory state (7). However, there is a paucity of data regarding the influence of obesity on the response to immune-based therapies. This gap in knowledge is particularly important given that 80% of EC-diagnosed women are obese (8) and immune checkpoint inhibitors (ICIs) are becoming a cornerstone for the treatment of EC (9).

Monoclonal antibodies blocking inhibitory checkpoints have recently changed the frontline treatment paradigm for advanced and recurrent EC. Dorstarlimab, a programmed cell death receptor-1 (PD-1) blocker, is now firstline therapy in conjunction with chemotherapy for patients with advanced EC based on results from the RUBY trial (10). Similarly, pembrolizumab, another PD-1 inhibitor, in combination with chemotherapy, has recently shown improved progression-free survival (PFS) in the frontline setting when compared with chemotherapy alone in the NRG-GY018 trial (11). In the second-line setting, ICI alone or in combination with the tyrosine kinase inhibitor (TKI) lenvatinib is FDA approved in recurrent EC after treatment with platinum-based chemotherapy in mismatch repair–deficient (MMR–deficient) and MMR-proficient EC, respectively (12, 13). Despite these clinical advances, there is a lack of validated clinical, molecular, and immunological biomarkers that can predict response to these therapies. To this end, one of the most intriguing findings in patients treated with ICI for non-EC malignancies is the “obesity paradox,” in which obese patients treated with ICI have improved outcomes compared with lean patients (14). Furthermore, higher BMI may also correlate with the rate of immune-related adverse events (irAEs) (15), suggesting that obesity might promote disruption of immune tolerance against both tumor and normal cells. While these observations have been described in a few solid tumors (16–20), the heterogeneity across different studies and the attenuation of these associations after adjusting for relevant clinical factors underscore the need for further investigation (18, 21). Importantly, this clinical association has yet to be explored in the context of EC.

Given the high prevalence of obesity in EC and the prominence of ICI in its management, this retrospective study aims to show whether obesity influences clinical outcomes in women with EC after treatment with ICI. By characterizing clinical markers for obesity, body fat distribution, and molecular EC subtypes, we found a strong association between overweight/obesity and improved clinical outcomes in EC patients treated with ICI alone or in combination with lenvatinib. Notably, this favorable prognostic impact remained independent of clinicopathological and molecular subtyping of EC. Additionally, after assessment of body fat distribution, we found that increased visceral adipose tissue (VAT) is particularly associated with the improved clinical outcomes observed in our cohort. Finally, obesity was also linked to elevated rates of irAEs after immunotherapy. Collectively, these findings highlight the role of increased adiposity in modulating the response to ICIs and their side effect profile in EC.

## Results

### Characteristics of patients with EC treated with ICI categorized by BMI.

**Figure 1:**
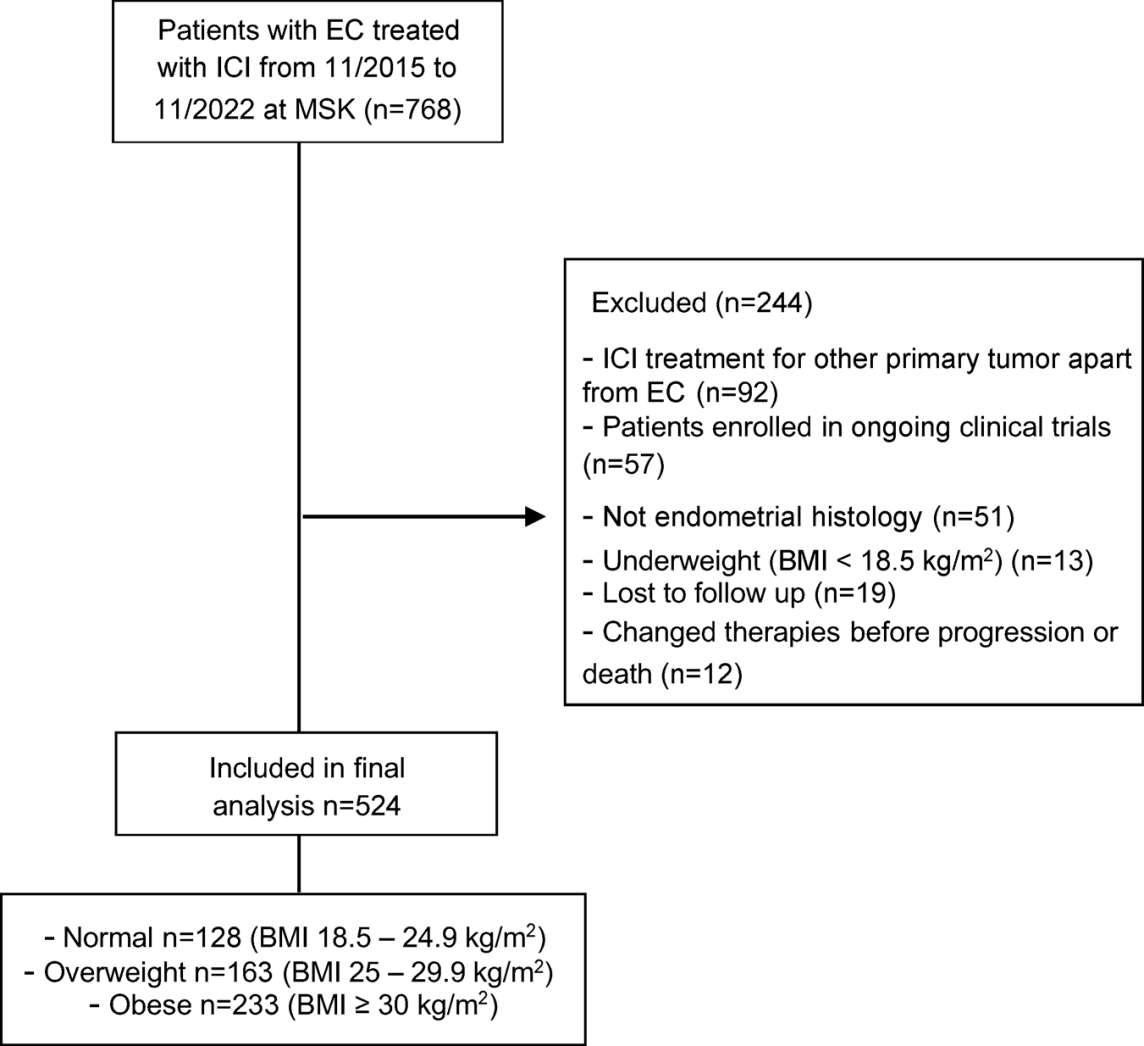
Consort diagram of the study population selection including exclusion criteria.

We retrospectively screened 768 patients diagnosed with EC that underwent treatment with ICI at MSK from November 2015 to November 2022. Out of these, 524 patients with recurrent, advanced, or metastatic EC were included in the final analysis (Figure 1). The main reason for exclusion was patients receiving ICI therapy to treat a non-EC malignancy. Underweight patients (BMI < 18.5 kg/m^2^) were also excluded from the analysis (Figure 1). The baseline clinical characteristics (at the start of ICI) of the patients included in the final analysis are shown in Table 1. Across the entire study cohort, the median age was 67 years (range 30–94), and the median BMI was 29.1 kg/m^2^. Most patients (85%) received anti–PD-1 therapy, while 15% received anti–PD-L1 therapy. Regarding the combination of ICI with other anticancer therapies, 307 patients (59%) were treated with pembrolizumab in combination with lenvatinib. The majority of patients received ICI therapy as a second (54%) or third line (27%) of treatment. Additionally, 437 patients (83%) underwent molecular subtyping, and 500 (95%) had a baseline CT for determination of fat distribution. When categorized by BMI before the start of ICI therapy, 128 patients (24%) had a normal BMI (18.5–24.9 kg/m^2^), whereas 163 (31%) were overweight (BMI 25–29.9 kg/m^2^) and 233 (44%) were obese (BMI ≥ 30 kg/m^2^). Except for self-reported race and age, no significant differences in baseline characteristics were observed among the BMI groups. The number of patients with elevated subcutaneous adipose tissue (SAT), VAT, and VAT/SAT ratio increased from normal BMI to overweight to obese patients (Table 1).

### Association between BMI and clinical outcomes after treatment with ICI in patients with EC.

**Figure 2:**
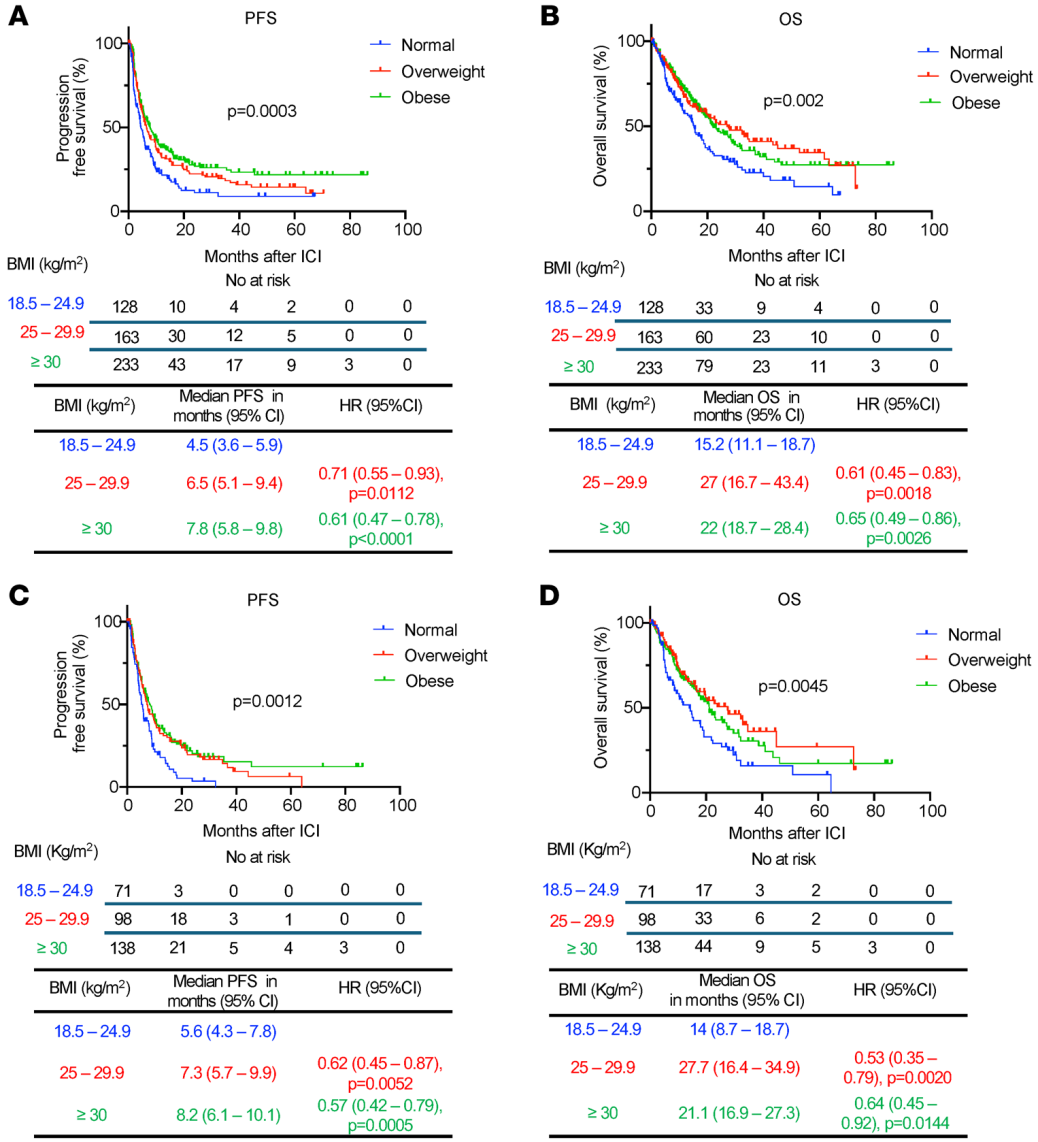
Survival outcomes of EC patients treated with ICI categorized by BMI. Kaplan-Meier curves for (**A**) PFS and (**B**) OS in patients with EC treated with ICI and categorized by BMI (normal: BMI 18.5–24.9 kg/m^2^ in blue; overweight: BMI 25–29.9 kg/m^2^ in red; obese: BMI >30 kg/m^2^ in green) (*n* = 524). Kaplan-Meier curves for (**C**) PFS and (**D**) OS in the subgroup of EC patients treated with the ICI pembrolizumab in combination with lenvatinib (*n* = 307). *P* values in the PFS and OS plots were calculated using a log-rank test. HRs and 95% CIs for overweight and obese patients were calculated using normal BMI as a reference.

First, we investigated whether an elevated BMI could influence the response to ICI in all EC patients included in the analysis. Survival analyses were performed after initiation of ICI therapy, revealing that patients categorized as overweight or obese exhibited a significantly prolonged PFS when compared with those with normal BMI after treatment with ICI (overweight versus normal BMI: median 6.5 versus 4.5 months, HR 0.71, 95% CI 0.55–0.93, *P* = 0.0112; obese versus normal BMI: median 7.8 versus 4.5 months, HR 0.61, 95% CI 0.47–0.78, *P* < 0.0001) (Figure 2A). Furthermore, patients with overweight and obesity demonstrated a significantly prolonged overall survival (OS) compared with patients with normal BMI after ICI (overweight versus normal BMI: median 27 versus 15.2 months, HR 0.61, 95% CI 0.45–0.83, *P* = 0.0018; obese versus normal BMI: median 22 versus 15.2 months, HR 0.65, 95% CI 0.49–0.86, *P* = 0.0026) (Figure 2B).

The combination of lenvatinib with the ICI pembrolizumab is the standard-of-care treatment for a substantial proportion of patients with MMR-proficient, advanced EC who have progressed after firstline platinum-based chemotherapy (12). As more than half of our cohort received this treatment combination (Table 1), we explored whether obesity was associated with clinical outcomes with this specific treatment regimen. Survival analyses in patients who received combination lenvatinib and pembrolizumab revealed that obese and overweight patients had significantly longer PFS (overweight versus normal BMI: median 7.3 versus 5.6 months, HR 0.62, 95% CI 0.45–0.87, *P* = 0.0052; obese versus normal BMI: median 8.2 versus 5.6 months, HR 0.57, 95% CI 0.42–0.79, *P* = 0.0005) and OS (overweight versus normal BMI: median 27.7 versus 14 months, HR 0.53, 95% CI 0.35–0.79, *P* = 0.0020; obese versus normal BMI: median 21.1 versus 14 months, HR 0.64, 95% CI 0.45–0.92, *P* = 0.0144) compared with patients with normal BMI (Figure 2, C and D).

**Figure 3:**
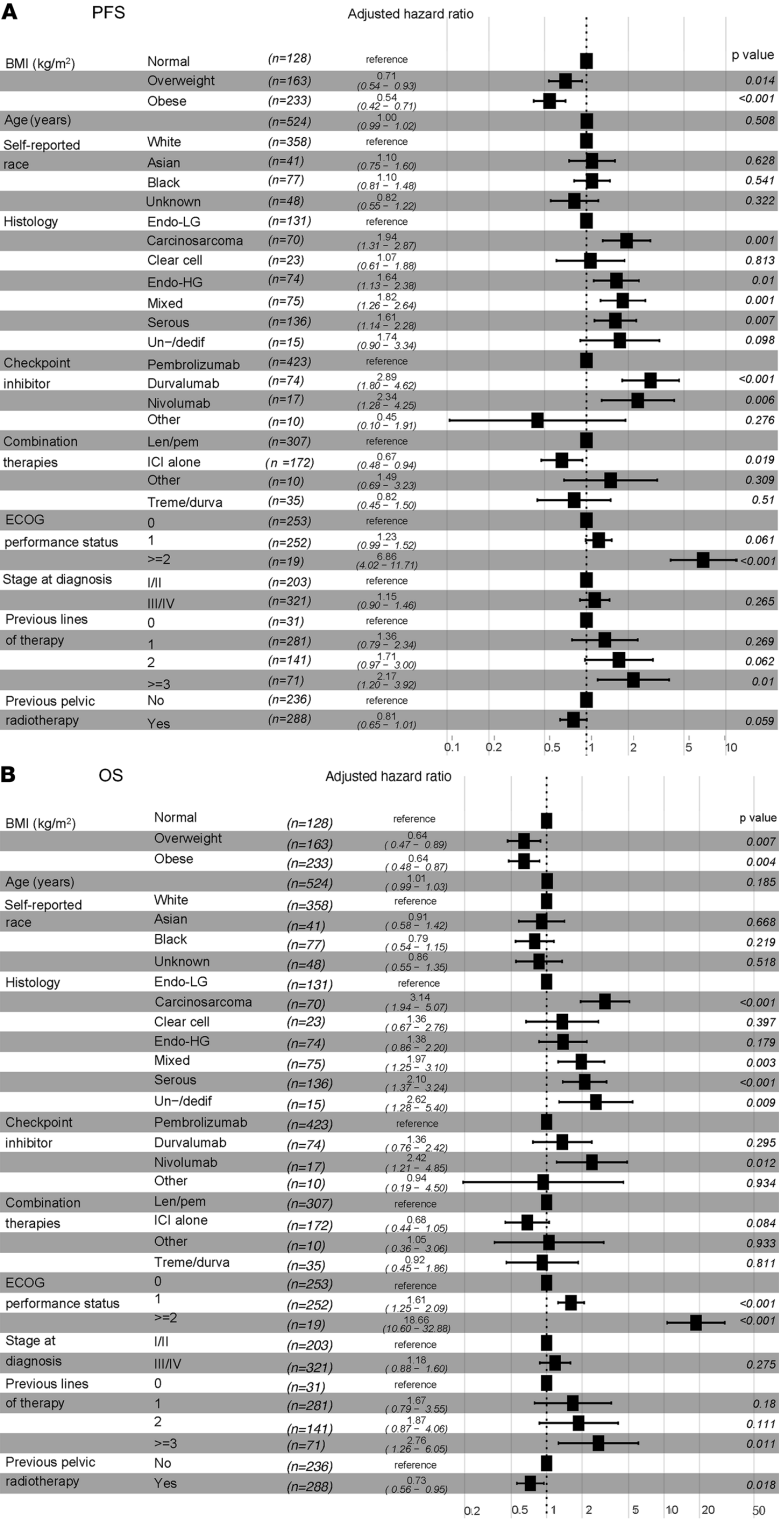
Multivariable Cox’s regression analysis of BMI and other clinical variables associated with response to ICI in EC patients. Forest plots of adjusted HRs and 95% CIs for patients with normal BMI (BMI 18.5–24.9 kg/m^2^) (reference group) compared with overweight (BMI 25–29.9 kg/m^2^) and obese (BMI > 30 kg/m^2^) for (**A**) PFS and (**B**) OS (*n* = 524) Analysis was adjusted for age, self-reported race, histology, checkpoint inhibitor treatment, combination therapies, baseline performance status, stage at diagnosis, prior lines of therapy, and previous pelvic radiotherapy. Endo-LG, endometrial low grade; Endo-HG, endometrial high grade; Un-/dediff, un/dedifferentiated; Len/pem, lenvatinib/pembrolizumab; Treme/durva, tremelimumab/durvalumab.

We then explored the impact of other baseline clinical variables on the PFS and OS of EC patients after treatment with ICI therapy. Similarly to what was shown with BMI, univariable Cox’s regression analysis demonstrated that specific histological types, stage at diagnosis, number of previous lines of therapy, and molecular subtype were significantly associated with changes in PFS and OS in EC patients treated with ICI (Table 2 and Table 3). Thus, we investigated whether BMI was independently associated with improved PFS and OS in our study cohort by controlling for these and other clinical variables. Multivariable Cox’s regression analysis demonstrated that baseline overweight and obese states were independently associated with improved PFS when compared with patients with normal BMI (overweight versus normal BMI: adjusted HR 0.71, 95% CI 0.54–0.93; obese versus normal BMI: adjusted HR 0.54, 95% CI 0.42–0.71) (Figure 3A). Similarly, overweight and obesity were independently associated with extended OS compared with normal BMI (overweight versus normal BMI: adjusted HR 0.64, 95% CI 0.47–0.89; obese versus normal BMI: adjusted HR 0.64, CI 95% 0.48–0.87) (Figure 3B). As expected, distinct histological types (carcinosarcoma, serous, un/dedifferentiated) and poor baseline Eastern Cooperative Oncology Group (ECOG) performance status (22) were independent predictors of worse PFS and OS. Overall, these results suggest a paradoxical association between elevated BMI and improved responses to ICI in patients with EC, further supporting BMI as an independent predictor of clinical response to ICI.

### Association between fat distribution and clinical responses to ICI in patients with EC.

While BMI serves as a well-established anthropometric indicator that is positively associated with cardiometabolic disease, it is important to recognize its inability to distinguish between fat and muscle mass (23). Furthermore, in the context of cancer, BMI may not precisely capture the association between AT and responses to distinct types of therapies (24). To address this limitation and assess whether specific fat distribution could predict clinical responses in patients with EC after ICI treatment, we performed 2D measurements of SAT and VAT at the level of L3/L4, which have shown a strong correlation with abdominal fat volumes and cardiometabolic risk factors (25). Out of the total cohort, 500 patients had available baseline CT scans to assess SAT and VAT areas.

**Figure 4:**
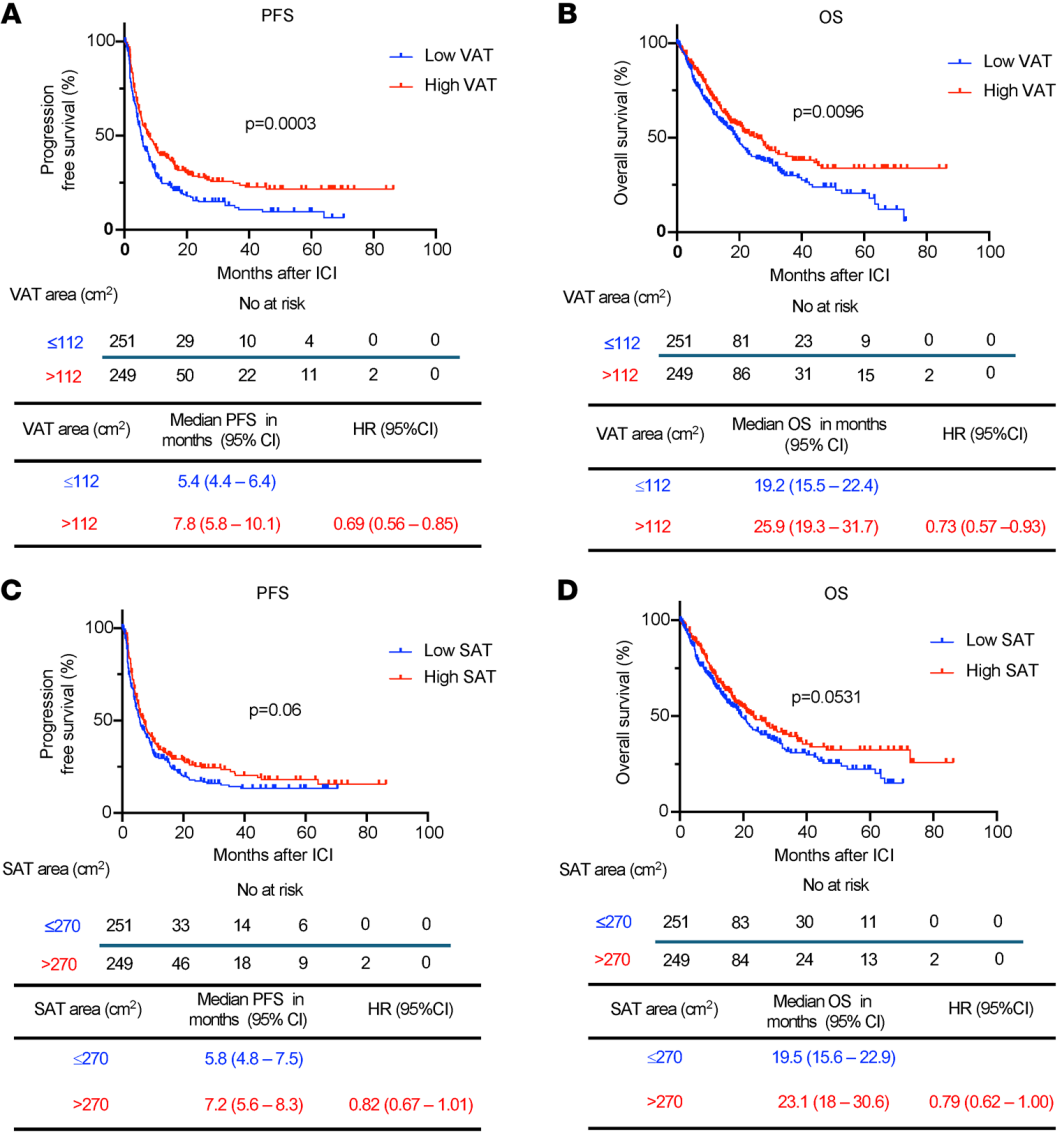
Survival outcomes after ICI in EC stratified by VAT and SAT area. Kaplan-Meier curves for (**A**) PFS and (**B**) OS in patients with EC following ICI treatment stratified by low and high VAT area (*n* = 500) (low VAT area: ≤112 cm^2^ in blue; high VAT area: >112 cm^2^ in red). Kaplan-Meier curves for (**C**) PFS and (**D**) OS in patients with EC following ICI treatment stratified by low and high SAT areas (low SAT area: ≤270 cm^2^ in blue; high SAT area: >270 cm^2^ in red) (*n* = 500). Patients were categorized as low or high VAT/SAT based on the median SAT and VAT of the entire cohort. *P* values in the PFS and OS plots were calculated using a log-rank test.

BMI correlated with both SAT (*r* = 0.79, *P* < 0.0001) and VAT areas (*r* = 0.71, *P* < 0.0001) (Supplemental Figure 1; supplemental material available online with this article; https://doi.org/10.1172/JCI180516DS1). We then categorized EC patients based on their median VAT (112 cm^2^) or SAT (270 cm^2^) area and examined their response to ICI, as previously performed in other studies (18). In patients with high VAT area, the median PFS after ICI was significantly prolonged compared with those with low VAT area (median 7.8 versus 5.4 months, HR 0.69, 95% CI 0.56–0.85, *P* = 0.0003) (Figure 4A). Furthermore, a high VAT area was associated with significantly prolonged OS compared with patients with low VAT area (median 25.9 versus 19.2 months, HR 0.73, 95% CI 0.57–0.93, *P* = 0.0096) (Figure 4B). In contrast, the relationship between SAT and survival outcomes was less pronounced. Among patients with EC and high SAT area, there was a numerically but not statistically significant improvement in PFS after ICI treatment compared with those with low SAT area (median 7.2 versus 5.8 months, HR 0.82, 95% CI 0.67–1.01, *P* = 0.06) (Figure 4C). Similarly, an elevated SAT area was numerically associated with prolonged OS compared with EC patients with a low SAT area (median 23.1 versus 19.5 months, HR 0.79, 95% CI 0.62–1, *P* = 0.0531) (Figure 4D). To further characterize the association between body-fat composition and clinical outcomes, we stratified VAT and SAT by quartiles. We found an incremental association between VAT area and PFS, but not OS, with patients in the highest quartile of VAT area showing a significant increase in PFS compared with patients in the lowest VAT area quartile (median 8.3 versus 5.7 months, HR 0.65, 95% CI 0.48–0.87, *P* = 0.004) (Supplemental Figure 2, A and B); in contrast, no association with PFS or OS was observed in the analysis of SAT area by quartiles (Supplemental Figure 2, C and D). Prior studies have suggested that the ratio between VAT and SAT could be a better predictor of cardiometabolic risk compared with VAT area measurement and BMI (26, 27). Hence, we determined the VAT/SAT ratio in our cohort and stratified patients in high and low VAT/SAT ratio according to the median (0.3723). Patients with a high VAT/SAT ratio exhibited a significant improvement in PFS (median 7.25 versus 5.5 months, HR 0.75, 95% CI 0.61–0.92, *P* = 0.0049), but not OS (Supplemental Figure 3, A and B). In a subgroup analysis performed in patients treated with lenvatinib and pembrolizumab (*n* = 296), we observed a trend toward both high VAT and SAT being associated with improved PFS, aligning with the significant results obtained in the larger cohort (Supplemental Figure 4, A–D).

To further interrogate VAT and SAT areas as independent predictors for the response to ICI in EC, we performed a multivariable Cox’s regression analysis to control for other relevant clinical variables (Supplemental Figures 5 and 6). High VAT area was independently associated with improved PFS (adjusted HR 0.73, 95% CI 0.59–0.91) following ICI treatment (Supplemental Figure 5A). High SAT was also found to be independently associated with prolonged PFS (adjusted HR 0.77, 95% CI 0.621–0.96), although this association was less profound (Supplemental Figure 6A). Neither high VAT nor high SAT was associated with OS (Supplemental Figure 5B and Supplemental Figure 6B). Overall, these results suggest that increased VAT (and to a lesser extent SAT) in obese patients may influence clinical responses to ICI in EC.

### Association of BMI and clinical responses after ICI across EC molecular subtypes.

Of the 524 patients in the total cohort, 437 (83%) had molecular subtyping performed using an integrated molecular-immunohistochemistry approach (28). The clinical characteristics of this subgroup of patients are shown in Supplemental Table 1. Within this cohort, 256 (59%) ECs were classified as copy number–high/*TP53*abnormal (CN-H/*TP53*abn), 97 (22%) as microsatellite instability–high (MSI-H), 81 (19%) as copy number–low/no specific molecular profile (CN-L/NSMP), and 3 (0.7%) as DNA polymerase epsilon (POLE) (Table 1 and Supplemental Table 1). Akin to previous reports (28, 29), MSI-H and *POLE* patients had higher PFS and OS compared with patients with CN-H/*TP53*abn and CN-L/NSMP in this set of EC patients treated with ICI (Supplemental Figure 7, A and B).

To determine whether BMI influenced responses to ICI across EC molecular subtypes, we built a separate multivariable Cox’s regression model in this subgroup accounting for molecular classification and clinicopathological features with *n* = 434 patients, excluding the POLE molecular subtype due to the small number of patients (*n* = 3). Overweight and obesity status remained independently associated with improved PFS (overweight versus normal BMI: adjusted HR 0.58, 95% CI 0.43–0.79; obese versus normal BMI: adjusted HR 0.53, 95% CI 0.4–0.71) and OS when compared with patients with normal BMI (overweight versus normal BMI: adjusted HR 0.5, 95% CI 0.35–0.72; obese versus normal BMI: adjusted HR 0.68, 95% CI 0.49–0.95). Additionally, ECOG performance status, specific histology types, and molecular subtype were confirmed to be independently associated with PFS and OS (Supplemental Figure 8, A and B).

**Figure 5:**
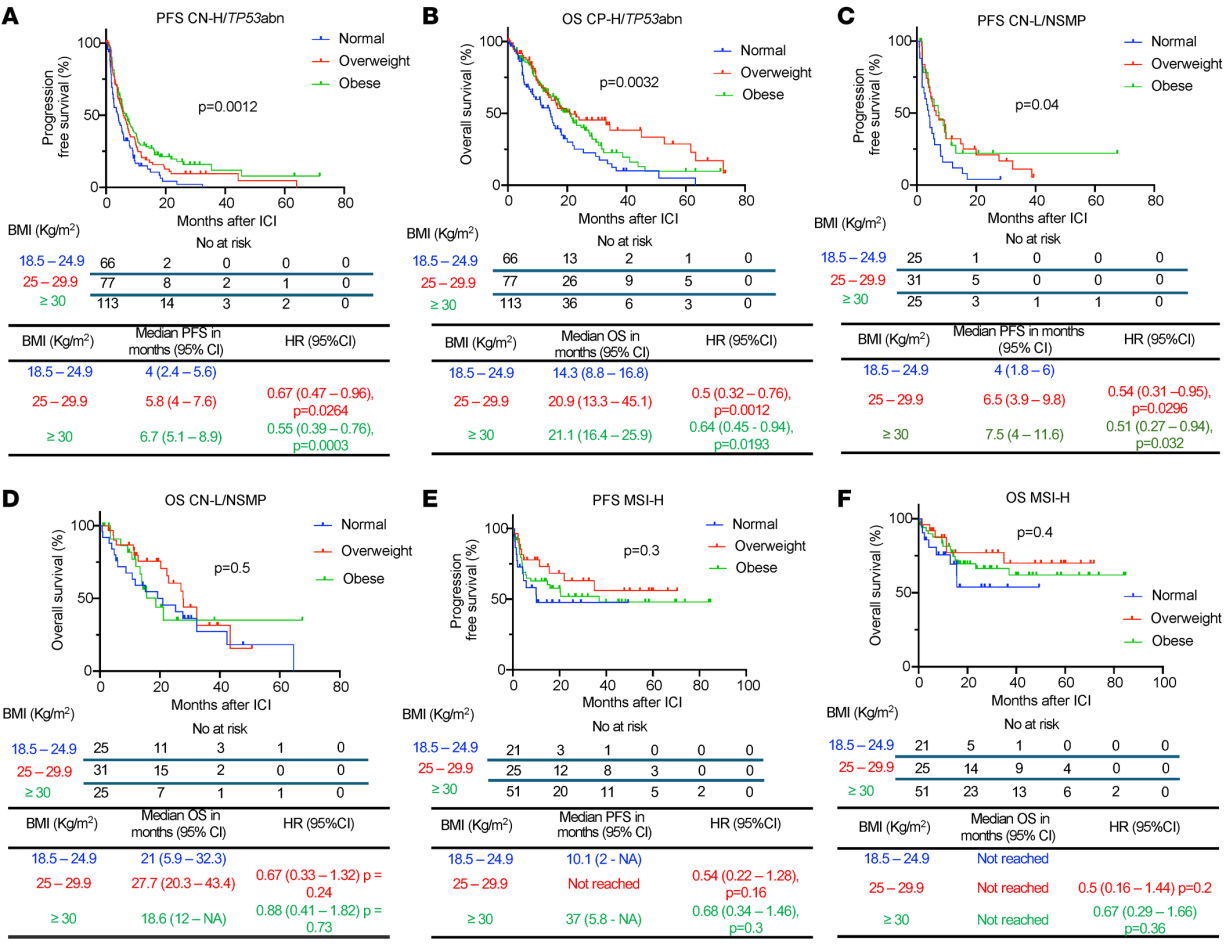
Survival outcomes following ICI in EC patients stratified by BMI across different molecular subtypes. Kaplan-Meier curves for (**A**) PFS and (**B**) OS in patients with CN-H/*TP53*abn EC following ICI treatment stratified by BMI (normal: BMI 18.5–24.9 kg/m^2^ in blue; overweight: BMI 25–29.9 kg/m^2^ in red; obese: BMI >30 kg/m^2^ in green) (*n* = 256). Kaplan-Meier curves for (**C**) PFS and (**D**) OS in patients with CN-L/NSMP EC following ICI treatment stratified by BMI (*n* = 81). Kaplan-Meir curves for (**E**) PFS and (**F**) OS in patients with MSI-H EC following ICI treatment stratified by BMI (*n* = 97). *P* values in the OS plots were calculated using a log-rank test. HRs and 95% CIs for overweight and obese patients were calculated using normal weight as a reference.

We then performed an exploratory subgroup analysis by molecular subtype class. In CN-H/*TP53*abn EC (*n* = 256), obese and overweight patients had significantly prolonged PFS (overweight versus normal BMI: median 5.8 versus 4.0 months, HR 0.67, 95% CI 0.47–0.96, *P* = 0.0264; obese versus normal BMI: median 6.7 versus 4.0 months, HR 0.55, 95% CI 0.39–0.76, *P* = 0.0003) and OS (overweight versus normal BMI: median 20.9 versus 14.3 months, HR 0.5, 95% CI 0.32–0.76, *P* = 0.0012; obese versus normal BMI: median 21.1 versus 14.3 months, HR 0.64, 95% CI 0.45–0.94, *P* = 0.0193) when compared with normal BMI patients after ICI (Figure 5, A and B). Regarding body fat distribution, among CN-H/ *TP53*abn EC patients with available baseline CT scan (*n* = 249), high VAT was associated with improved PFS (median 6.86 versus 5.18 months. HR 0.68, 95% CI 0.51–0.89, *P* = 0.0047), but not OS (Supplemental Figure 9, A and B). High SAT was also associated with improved PFS (median 5.93 versus 5.25 months. HR 0.75, 95% CI 0.57–0.99, *P* = 0.0441) and OS (19.86 versus 15.96 months, HR 0.72, 95% CI 0.52–0.99, *P* = 0.0449) (Supplemental Figure 9, C and D). In CN-L/NSMP EC (*n* = 81), obese and overweight patients had a significantly prolonged PFS to ICI compared with individuals with normal BMI (overweight versus normal BMI: median 6.5 versus 4 months, HR 0.54, 95% CI 0.31–0.95, *P* = 0.0296; obese versus normal BMI: median 7.5 versus 4 months, HR 0.51, 95% CI 0.27–0.94, *P* = 0.032) (Figure 5C) with no differences in OS (Figure 5D). Body fat distribution was also assessed in CN-L/NSMP patients with available CT scan (*n* = 79). Patients with high VAT had a trend toward improved PFS (median 7.46 versus 4.59 months, HR 0.62 95% CI 0.38–1.01, *P* = 0.0525), but not OS (Supplemental Figure 10, A and B). High SAT was not associated with either improved PFS or OS (Supplemental Figure 10, C and D). Finally, no differences in PFS or OS were observed in MSI-H EC across BMI categories (*n* = 97) or VAT/SAT area categories (*n* = 90) (Figure 5, E and F, and Supplemental Figure 11, A–D). Overall, our data underscore the impact of obesity and overweight on prognosis, independently of clinicopathological and molecular factors. Moreover, our analyses suggest that these relationships are particularly profound in patients with CN-H/*TP53*abn EC.

### Association between BMI and irAEs in EC patients treated with ICI.

**Figure 6:**
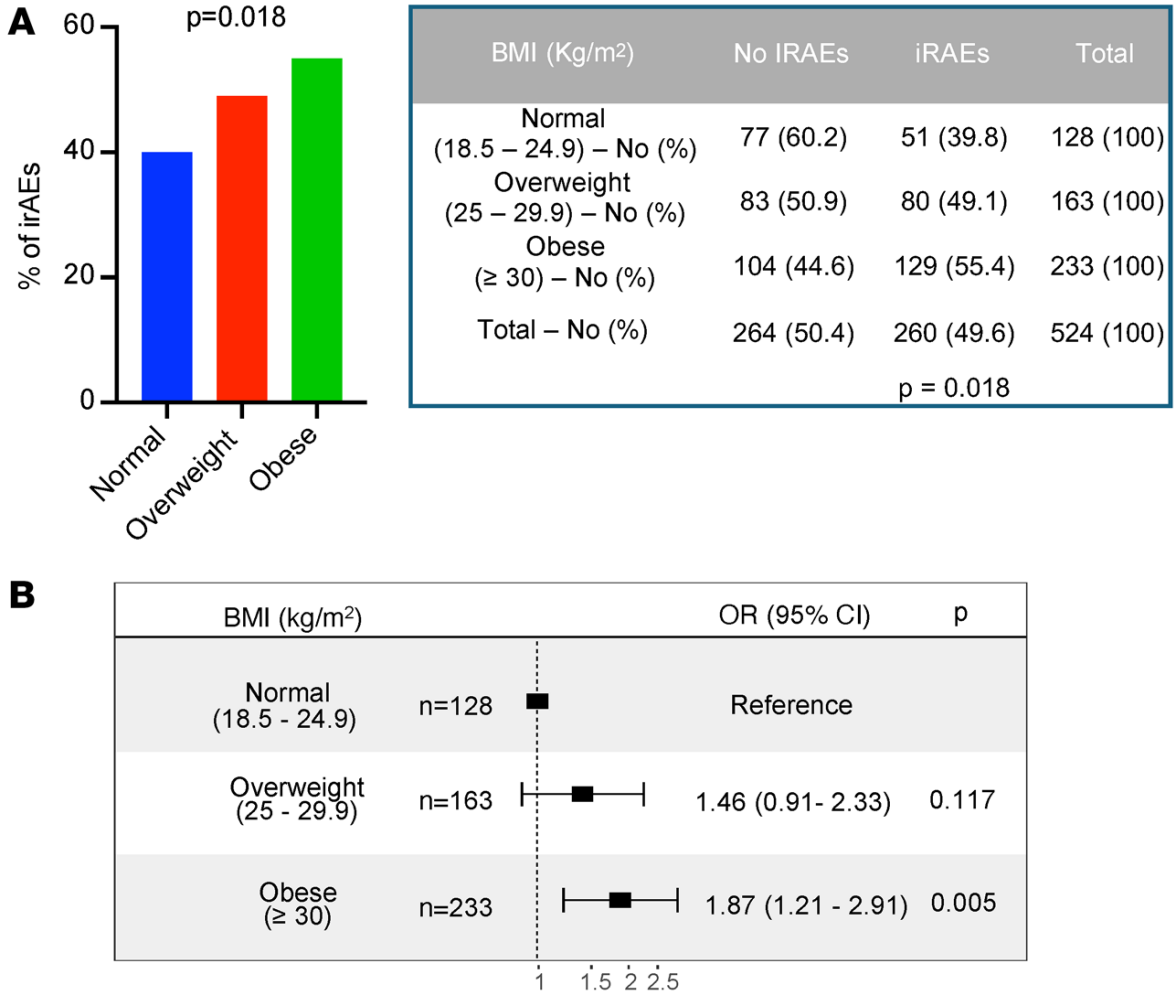
Incidence of irAEs in EC patients after treatment with ICI stratified by BMI. (**A**) Percentages and absolute numbers of irAEs across BMI categories (normal: BMI 18.5–24.9 kg/m^2^ in blue; overweight: BMI 25–29.9 kg/m^2^ in red; obese: BMI >30 kg/m^2^ in green). Representative figure (left) and table (right) are shown. *P* value in the bar graph and table was calculated using χ^2^ test. (**B**) Forest plot of ORs and 95% CIs for patients with normal BMI (reference) compared with overweight and obese patients and their incidence of irAEs.

irAEs are autoimmune conditions affecting any organ in the body after ICI administration, with heterogeneous clinical presentations and poorly understood underlying biology (30). Previous studies suggest a positive association between improved clinical responses to ICI and development of irAEs (31–34). We investigated whether BMI is associated with the frequency of irAEs (assessed by Common Terminology Criteria for Adverse Events [CTCAE] version 5; https://ctep.cancer.gov/protocolDevelopment/electronic_applications/ctc.htm) after ICI treatment. In the total cohort, the rate of irAEs of any grade was 49.6%. BMI category was significantly associated with the incidence of iRAEs (*P* = 0.018) (Figure 6A). More specifically, obesity, but not overweight, was associated with increased odds of developing irAEs after ICI therapy (overweight versus normal BMI: OR 1.46, 95% CI 0.91–2.33; obese versus normal BMI: OR 1.87, 95% CI 1.21–2.91) (Figure 6B). We also analyzed the incidence of irAEs in patients with high versus low VAT and SAT and did not find significant differences (Supplemental Figure 12, A and B). To further characterize the link between BMI and irAEs, we stratified irAEs based on their severity (assessed by CTCAE criteria, version 5) and analyzed whether BMI, VAT area, or SAT area was positively associated with severe adverse events. No significant differences were found in the proportion of mild/moderate (G1/G2) versus severe (G3/G4/G5) irAEs when stratified by BMI category or high/low VAT and SAT (Supplemental Figure 12, C–E). There was a trend toward an association between severe irAEs and BMI categories (*P* = 0.0523).

We then interrogated whether BMI influenced the incidence of distinct irAEs. In the whole cohort, thyroid irAEs were the most reported events (34% hypothyroidism and 14% hyperthyroidism) (Table 4). Next in prevalence were gastrointestinal (colitis, hepatitis, pancreatitis) (11%), skin (6%), and rheumatoid (2%) irAEs (Table 4). Other organ systems had fewer than 10 cases reported for the whole cohort (Supplemental Table 2). When stratified by BMI, obese patients had a numerically higher rate of hypothyroidism compared with those with normal BMI (normal BMI: 27%; overweight: 33%; obese 39%; *P* = 0.1) (Table 4); no differences in other irAE were observed across BMI categories.

### Exploratory analysis of baseline circulating WBCs in EC patients treated with ICI.

**Figure 7:**
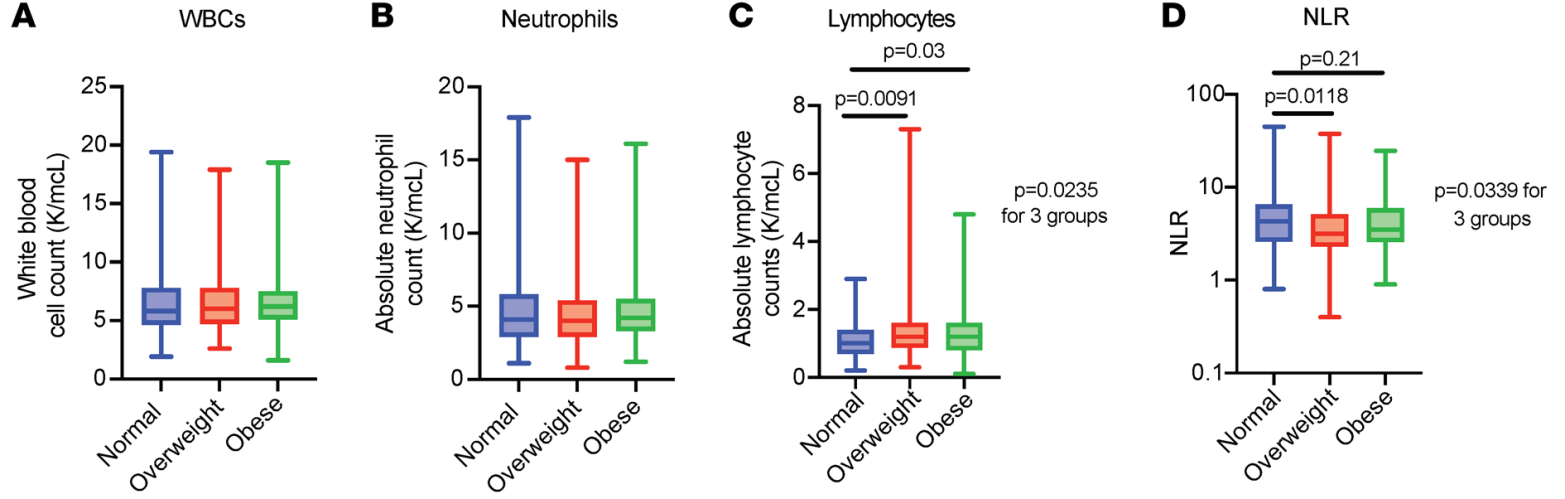
WBC, neutrophil, and lymphocyte counts in EC patients treated with ICI stratified by BMI. Number of (**A**) WBCs, (**B**) neutrophils, (**C**) lymphocytes, and (**D**) calculated NLR across BMI categories (normal: BMI 18.5–24.9 kg/m^2^ in blue; overweight: BMI 25–29.9 kg/m^2^ in red; obese: BMI >30 kg/m^2^ in green). *P* values comparing 2 groups were calculated using the Mann-Whitney *U* test; *P* values comparing 3 groups were calculated using the Kruskal-Wallis test.

To investigate the potential mechanism behind the protective effect of overweight and obesity in patients with EC treated with ICI, we performed an exploratory analysis using the baseline levels of circulating WBCs as a proxy for systemic inflammation. All the patients in our cohort (*n* = 524) had a baseline WBC count and neutrophil count (before ICI treatment), whereas 451 had baseline lymphocyte counts. We found that there were no differences between numbers of WBCs and neutrophils across BMI categories (Figure 7, A and B). In contrast, we found that there was a higher number of absolute lymphocytes in overweight and obese patients with EC before ICI treatment (Figure 7C). We then calculated the neutrophil-to-lymphocyte ratio (NLR), which has been proposed as a surrogate marker of inflammation status and adaptive immune surveillance (35). Furthermore, low NLR has been associated with improved outcomes to ICI in pan-cancer cohorts (35). There was a significant difference in NLR across BMI categories (*P* = 0.0339); overweight patients had a significantly lower NLR compared with normal BMI (*P* = 0.0118), with no differences found between obese and normal BMI categories (Figure 7D). These data point toward a potential role of circulating immune cells in mediating the association between elevated BMI and improved clinical outcomes in EC patients after ICI therapy.

## Discussion

In this study, we demonstrate that overweight and obese patients with EC exhibit significantly prolonged survival following treatment with ICI compared with patients with normal BMI. Importantly, these associations remained significant after adjusting for relevant clinical factors and EC molecular subtypes. Moreover, elevated adiposity, especially in the visceral compartment, independently predicts improved PFS. Importantly, molecular classification of EC highlights that the association between obesity and response to ICI is particularly pronounced in patients with the CN-H/*TP53*abn EC subtype and is absent in patients with MSI-H EC. Finally, obesity was also associated with a higher rate of irAEs after ICI in EC patients, suggesting an enhanced immune response in this setting.

The “obesity paradox” has been investigated in other cancer types after treatment with ICI (16–20), with a first study in metastatic melanoma revealing improved survival outcomes in male obese patients receiving ICI or targeted therapy, but not in patients receiving chemotherapy (16). While this association persisted after adjusting for other clinical factors, the findings were limited to BMI categories, and other markers for obesity in this cohort were not explored. A separate study found a positive correlation between BMI and response to atezolizumab in non–small cell lung cancer (20), but no correlation was seen with the development of irAEs. In contrast, a subsequent study in patients with renal cell carcinoma showed no association between obesity and response to ICI after adjusting for other clinical variables (18). Collectively, these results suggest that obesity may have a different effect on responses to ICI depending on the type of malignancy and underscore the need for tumor-specific studies to better understand these interactions.

In line with our results, a pan-cancer study indicated that obesity and overweight status were associated with improved PFS and OS after ICI therapy, with a suggestive trend in a small subgroup of EC patients (19). Our study expands on these observations and uniquely establishes the positive correlation of obesity and elevated adiposity in EC patients with improved responses to ICI. After adjusting for multiple factors, including tumor molecular subtyping, obesity remained an important predictor of improved clinical outcomes. Furthermore, our analyses included assessment of various body composition parameters beyond BMI, revealing an association between elevated VAT and favorable outcomes. Importantly, our cohort is racially and ethnically diverse, which makes our findings applicable to the real-world setting.

The mechanisms underlying the effects of obesity and AT dysfunction on immune responses during ICI treatment remain largely underexplored. In preclinical models for melanoma and lung, colorectal, and breast cancer, obese mice exhibit accelerated tumor growth and progression when compared with lean mice (36–38). These effects are partially attributed to an exhausted PD-1^hi^CD8^+^ T cell phenotype or a general decrease in CD8^+^ T cell infiltration (36–38). Interestingly, responses to PD-1 blockade were different across these tumor models. In melanoma and lung cancer models, PD-1 blockade reinvigorated PD-1^hi^CD8^+^ T cells, resulting in enhanced antitumor activity in obese but not in lean mice (36). Of note, this T cell–exhausted phenotype was partially mediated by leptin, highlighting a potential crosstalk between AT and immune responses to cancer. Conversely, PD-1 blockade did not confer additional benefit in obese mice with colorectal or breast cancer (37). Additional correlative studies in human endometrial tumor samples showed that CD8^+^ T cells and PD-L1 expression were decreased in the tumor microenvironment of patients with elevated BMI. However, PD-1, the main marker for T cell exhaustion, was not measured directly in this study. We hypothesize that obesity in EC may induce a dysfunctional CD8^+^ T cell phenotype with elevated expression of PD-1 and other inhibitory immune checkpoints. As a result, this exhausted phenotype might be more responsive to “reinvigoration” by anti–PD-1 therapy and other immunotherapies. Further prospective studies analyzing PD-1 expression in T cells from the EC tumor microenvironment are warranted to confirm this hypothesis. To this end, we did find an increased number of circulating lymphocytes and a lower systemic NLR in patients with overweight and obesity in our study, highlighting a role for potential circulating immune cells in mediating this “obesity paradox.” Of note, about half of the patients in our study received treatment with the combination of the PD-1 blocker pembrolizumab and the TKI lenvatinib, raising the question of whether this combination treatment could have a unique effect over immune responses in the context of obesity and EC. Studies analyzing the EC tumor microenvironment before and after ICI therapy and its association with circulating inflammatory factors and AT inflammation are crucial for fully dissecting these mechanisms.

Our study also highlights the association between fat mass, specifically VAT, and enhanced responses to ICI therapy. These findings contrast with previous studies that identified elevated VAT as an adverse prognostic factor in patients with EC (39, 40). Transcriptomic analysis of omental VAT from women with EC revealed that patients with higher AT inflammation exhibited increased expression of genes associated with proinflammatory pathways, which may result in increased susceptibility to ICI (41). Overall, elevated body weight and AT inflammation seem to contribute to a dysfunctional immune response in EC, promoting cancer growth. Paradoxically, we hypothesize that this dysregulated immune state might confer susceptibility to ICI therapy, resulting in a protective effect in patients with obesity and increased visceral adiposity.

EC is a clinically, histologically, and molecularly heterogeneous disease. The EC molecular classification holds prognostic value (42, 43), and in certain instances, it offers predictive value into specific cancer therapies (44–46). Our study reveals that baseline BMI is a predictor of response to ICI independently of the molecular classification. Notably, in a subgroup analysis, patients with CN-H/*TP53*abn EC displayed a particularly strong association between elevated BMI and improved ICI outcomes. This is relevant, as patients with this molecular subtype have the worst clinical outcomes (29), emphasizing the unmet need for biomarkers predicting clinical responses in this group. Furthermore, evidence suggests differences in the immune microenvironment across different EC molecular subtypes (47–49). For instance, the *TP53* mutant subtype exhibits the highest densities of both PD-1^+^ T cells and PD-L1^+^ macrophages compared with other molecular subtypes (48). Understanding how genetic alterations in EC shape the tumor immune microenvironment and influence therapy responses will address a critical knowledge gap. Prospective studies investigating these relationships across different molecular subtypes are essential for validating our findings.

Finally, our studies reveal an association between obesity and higher rates of irAEs. Most of the adverse events reported were thyroid-immune related, likely linked to the prevalent use of lenvatinib in our cohort (12). Of note, we found a trend toward increasing incidence of severe irAEs with BMI categories; mechanistically, it remains unclear whether the higher responses to ICI in patients with elevated BMI contribute to the higher incidence of mild/moderate irAEs. Increased T cell activation and proliferation in response to ICI, secretion of systemic proinflammatory cytokines, and crossreactivity in tumoral antigenicity have been suggested as potential mediators of these irAEs (50).

Our study has several limitations. First, its retrospective design highlights the need for prospective studies for further validation. However, we controlled for multiple clinical confounders, including molecular subtyping, which solidifies our findings. Second, not all the patients had available molecular characterization and baseline CT scans for body composition assessment, yet subset analysis on corresponding patients yielded results consistent with the total cohort. Finally, baseline BMI may not fully reflect weight dynamics in EC patients before and after ICI treatment, leading to potential bias in our analysis (51). To address this, we chose to complement our analyses with alternative body composition measurements, which aligned with the results from the BMI analysis.

In conclusion, our study presents clinical responses to ICI from a large cohort of EC patients stratified by BMI. Obesity and overweight were independently associated with improved survival after ICI, particularly in high-risk molecular subtypes of EC. Visceral fat mass, notably, was predominantly associated with these improved clinical responses, suggesting a potentially unique role in mediating effective immune responses in EC. Overall, our findings underscore the need for further mechanistic studies using EC biospecimen analysis and relevant EC preclinical models.

## Methods

### Sex as a biological variable.

Sex was not considered as a variable given the disease etiology.

### Cohort characteristics.

To screen for eligible patients, we extracted available electronic health record data from all patients with a histological diagnosis of EC that received treatment with ICI at MSK from November 2015 to November 2022 (*n* = 768). We included patients who received at least 1 dose of ICI and had advanced, recurrent, or metastatic EC. We then excluded patients based on the criteria outlined in Figure 1 as follows: patients who received ICI therapy to target a primary tumor different from EC, those with non-EC histology, underweight patients as defined by BMI of less than 18.5, patients who received 1 dose of ICI and were subsequently lost to follow-up (changed providers from MSK to another health institution), patients who received other anticancer therapy after ICI had been started before evidence of progression or death, and patients enrolled in ongoing clinical trials. Baseline patient characteristics (before ICI treatment), including age, BMI, self-reported race, previous lines of therapy, and ECOG performance status, were obtained by manual chart review and used for subsequent analysis. BMI was categorized according to World Health Organization criteria as normal (18.5–24.9 kg/m^2^), overweight (25–29.9 kg/m^2^), and obese (≥30 kg/m^2^). Stage at diagnosis was defined by the The International Federation of Gynecology and Obstetrics (FIGO) 2009 classification (52).

Given that the combination of pembrolizumab and lenvatinib was the most common treatment regimen in our cohort, we performed a subgroup analysis of survival outcomes in these patients (*n* = 307). We additionally performed survival analysis stratified by AT area in patients with available baseline CT scans (a maximum of 3 months before ICI initiation) (*n* = 500). Those patients with no available baseline CT scan were excluded in this subgroup analysis. For survival analysis stratified by molecular subtype and BMI, we analyzed the subgroup of patients with these data available (*n* = 437).

### Outcomes.

Available clinical records were reviewed for the primary study outcomes. PFS was defined as the time from first ICI infusion to disease progression or any cause of death; patients without progression were censored at date of last office visit. OS was defined as the time from first ICI infusion to any cause of death; patients who did not die were censored at date of last office visit. Progression was assessed using the Response Evaluation Criteria in Solid Tumors (RECIST), version 1.1 (53). When formal RECIST evaluation was not available (*n* = 365, 68%), we manually reviewed physicians’ notes and imaging reports to classify overall best response using the same criteria. For consistency, all patients were reviewed by the same investigator and supervised by a senior author.

irAEs were defined according to the CTCAE, version 5, by manual review of the chart. Thyroid-related adverse events were the most common in the cohort and were further divided into hypo- and hyperthyroidism. irAEs included specific ones, such as colitis, pneumonitis, hepatitis, pancreatitis, nephritis, and myocarditis. Grouped irAEs were skin (maculopapular eruptions, dermatitis, pruritus), rheumatoid (arthritis, myositis, polymyalgia rheumatica), other endocrine (diabetes, hypophysitis, adrenal insufficiency), neurological (encephalitis, meningitis), ocular (uveitis, optic neuritis), and hematologic (hemolytic anemia).

### Measurement of body fat distribution.

Body fat composition variables were assessed using commercially available software (Aquarius iNtuition, version 4.4.13.P6.; TeraRecon). Outer abdominal circumference and SAT and VAT areas (CT density range: –195–45 Hounsfield units) were semiautomatically calculated from pretreatment CT scans, using the axial plane at the L3/L4 intervertebral level (24). In cases with incorrect delimitation of the SAT and VAT, the radiologist manually fixed its limits using visual assessment and recalculated these variables. Based on the median of SAT area (270 cm^2^), patients were further categorized as low SAT (≤270 cm^2^) and high SAT areas (>270 cm^2^). Based on the median VAT area (112 cm^2^), patients were further categorized as low VAT area (≤ 112 cm^2^) and high VAT area (>112 cm^2^). Patients were additionally categorized in quartiles based on SAT and VAT areas. For SAT, quartile 1 was 3–189 cm^2^, quartile 2 was >189–270 cm^2^, quartile 3 was >270–380 cm, and quartile 4 was >380–866 cm^2^. For VAT, quartile 1 was 4.5–56.1 cm^2^, quartile 2 was >56.1–112 cm^2^, quartile 3 was >112–172 cm^2^, and quartile 4 was >172–470 cm^2^. When developing subgroup analyses, high VAT and SAT areas were based on the median for each particular subgroup.

### Clinicopathologic features.

Pathology reports authored by departmental gynecologic pathologists throughout the study time frame were reviewed. These contained histopathologic data evaluated through a uniform diagnostic approach with biweekly diagnostic consensus conferences, as previously described (54). Histologic type, FIGO 2009 stage, and endometrioid tumor grade were recorded based on the patient’s initial pathologic diagnosis, as previously described (28). All histologic subtypes were included (i.e., endometrioid, serous, clear cell, carcinosarcoma, un/dedifferentiated, and mixed/high-grade not otherwise specified (NOS). The highest histologic grade for endometrioid type ECs was recorded from either the preoperative biopsy or hysterectomy specimen.

### Molecular subtype classification.

Molecular subtype using an integrated molecular-immunohistochemistry approach was determined as previously described (28). In brief, for cases with a minimum tumor purity of 20%, (a) POLE molecular subtype was defined by the presence of a known POLE hot spot exonuclease domain mutation (55), (b) MSI-H molecular subtype was assigned if the MSIsensor score was 10 or more (56) and/or if the tumor sample was MMR deficient (MMRd) based on IHC MLH1, MSH2, and/or PMS2 and MSH6, (c) CN-H/*TP53*abn molecular subtype was assigned based on the presence of a TP53 homozygous deletion or a pathogenic driver mutation, and (d) CN-L/NSMP molecular subtype was assigned if a tumor sample did not harbor any of the defining features of the other 3 subtypes.

### WBC quantification.

Absolute WBC, neutrophil, and lymphocyte numbers were gathered from complete blood counts collected up to 4 weeks prior to ICI treatment. NLR was calculated as the absolute count of neutrophils divided by the absolute count of lymphocytes.

### Statistics.

Kruskal-Wallis test was used to compare continuous variables across 3 BMI categories and Mann-Whitney *U* test for comparing 2 groups. The χ^2^ or Fisher’s exact test was used for categorical variables. PFS and OS curves were plotted using the Kaplan-Meier method, and log-rank test was used to compare survival distributions. HRs were estimated using Cox’s proportional hazards model. Multivariable Cox’s regression models included BMI category, VAT or SAT group, and clinically relevant variables as covariates. ORs were calculated using logistic regression. HR and OR estimates are reported with 95% CIs and corresponding *P* values. Spearman’s correlation coefficient was used to assess the linear relationship between continuous BMI and VAT or SAT area. All statistical analyses were performed using SAS Studio, version 3.81, and R, version 4.0.4. A *P* value of less than 0.05 was considered significant.

### Study approval.

The institutional review boards at MSK approved this retrospective study. All patients provided written, informed consent for tumor genomic sequencing.

### Data availability.

All data generated in this study are included in the article, supplemental material, or Supporting Data Values file or can be obtained upon request.

## Author contributions

NGB and JCO conceived and designed the study. NGB, WAZ, CS, and CD collected the clinical data. EJO, JG, AMP, and AJP performed the radiological analysis. NGB and CSJ conducted the statistical analysis. JCO, PC, JJM, CA, BW, and VM supervised data collection and reviewed and revised the manuscript. PC and JCO supervised the study. CD, BW, and JJM performed the molecular subtype analysis. NGB and JCO wrote the original draft. All authors contributed to manuscript preparation.

## Supplementary Material

Supplemental data

ICMJE disclosure forms

Supporting data values

## Figures and Tables

**Table 3:**
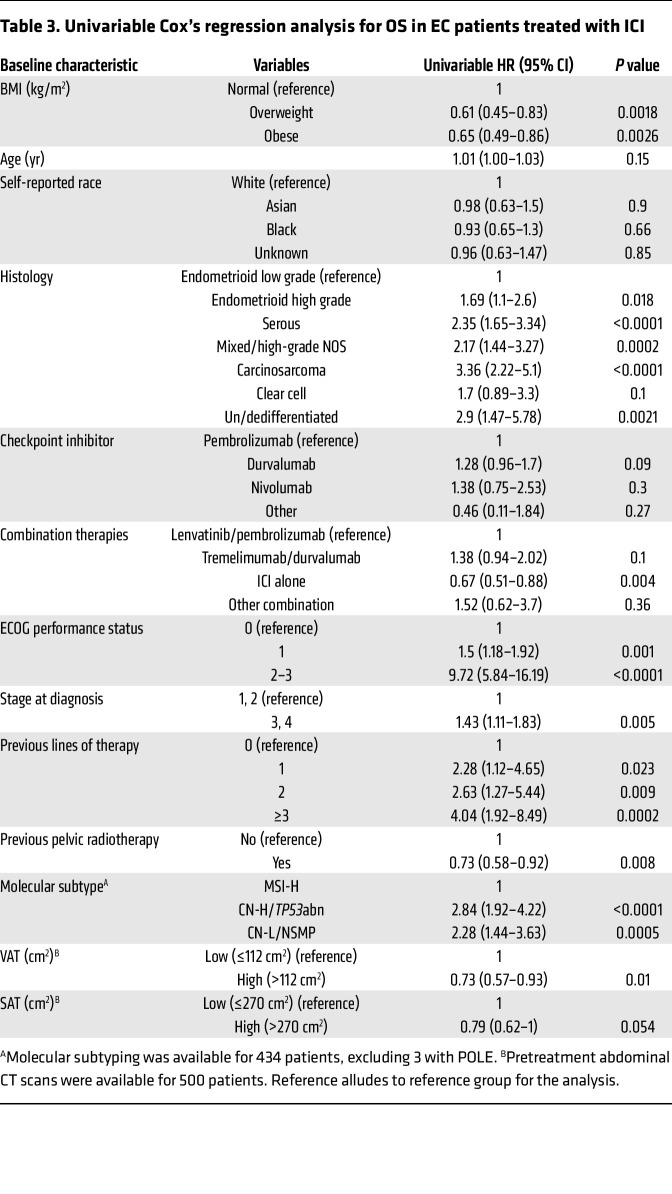
Univariable Cox’s regression analysis for OS in EC patients treated with ICI

**Table 2:**
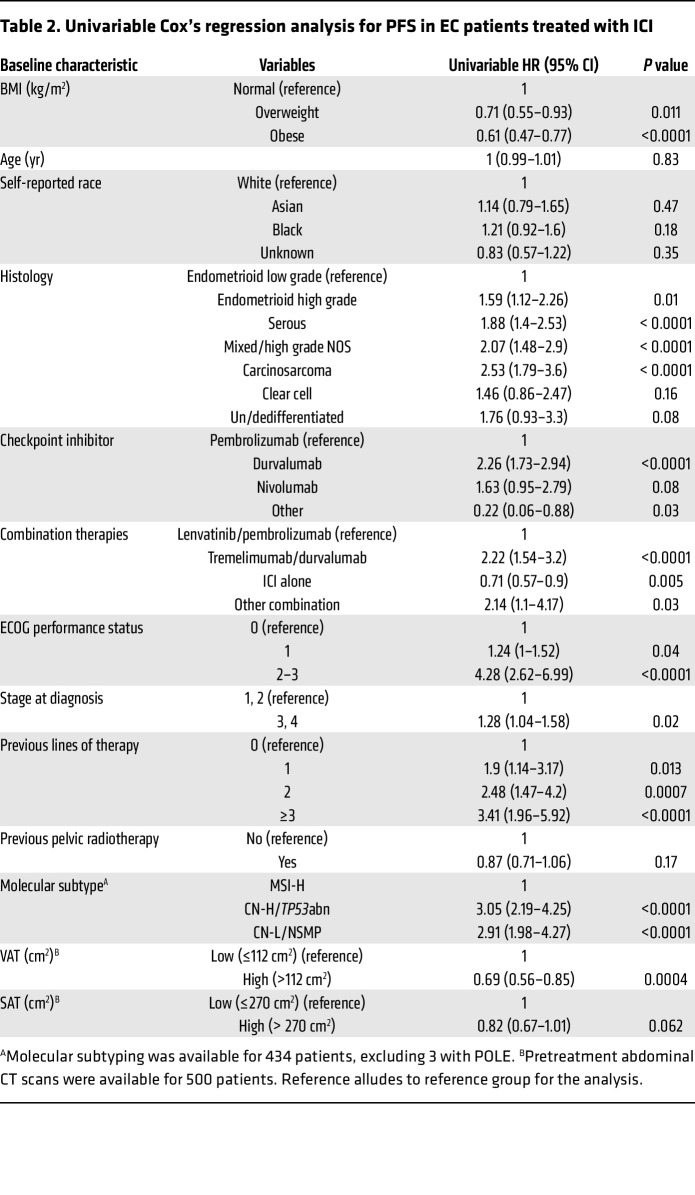
Univariable Cox’s regression analysis for PFS in EC patients treated with ICI

**Table 1:**
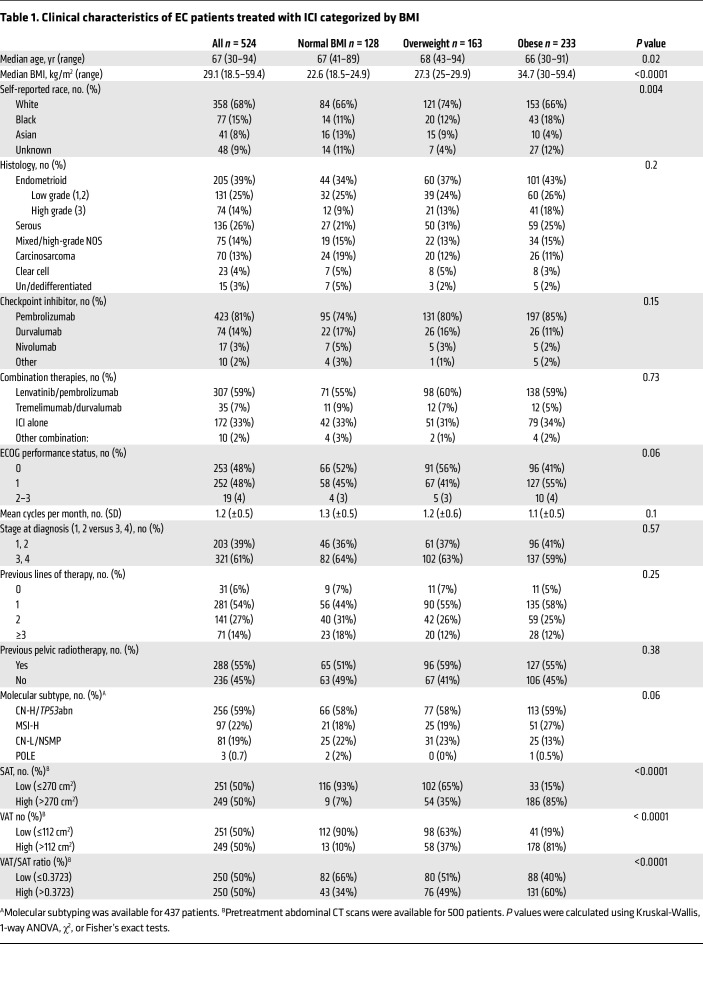
Clinical characteristics of EC patients treated with ICI categorized by BMI

**Table 4:**
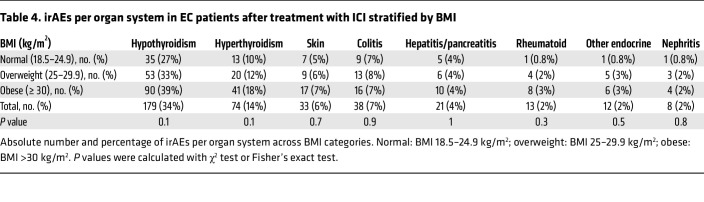
irAEs per organ system in EC patients after treatment with ICI stratified by BMI

## Version 1 06/20/2024

In-Press Preview

## Version 2 09/03/2024

Electronic publication
