# Supplementary material for: Body mass index and adiposity influence responses to immune checkpoint inhibition in endometrial cancer
Source: J Clin Invest. 2024 Jun 20;134(17):e180516. doi: 10.1172/JCI180516 (PMC11364395; doi:10.1172/JCI180516)
Supplement: Supplemental data [file jci-134-180516-s146.pdf]

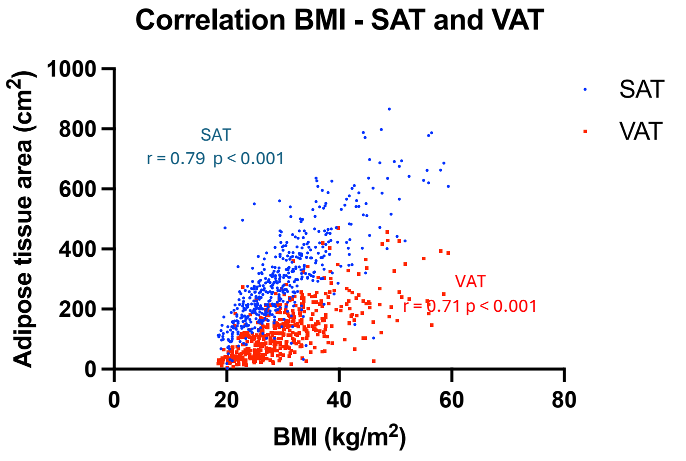

Supplemental Figure 1. Correlation between BMI and VAT or SAT area. Scatterplots depicting the correlation between BMI and VAT and SAT area. Correlation was assessed by Spearman's rank correlation coefficient n=500. BMI, body mass index; VAT, visceral adipose tissue; SAT, subcutaneous adipose tissue.

A. B.

EC patients treated with ICI stratified by VAT area (quartiles)

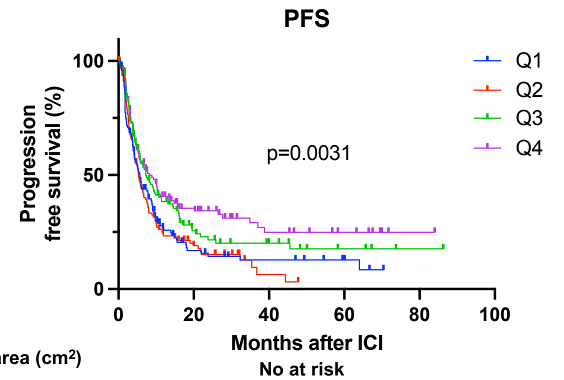

VAT area (cm^2)	No at risk					
(4.5 – 56.1)	125	14	8	4	0	0
(>56.1 – 112)	126	15	2	0	0	0
(>112 – 172)	124	20	10	4	1	0
(>172 – 470)	125	30	12	7	1	0

VAT area (cm^2)	Median PFS in months (95% CI)	HR (95%CI)
Q1 (4.5 – 56.1)	5.7 (4.3 - 7.9)	
Q2 (>56.1 – 112)	5.3 (4.1 - 6.7)	1.05 (0.79 - 1.38), p=0.75
Q3 (>112 –172)	7.3 (5.7 - 10.7)	0.76 (0.57 – 1.01), p=0.06
Q4 (>172 – 470)	8.3 (5.4 - 11.1)	0.65 (0.48 – 0.87), p=0.004

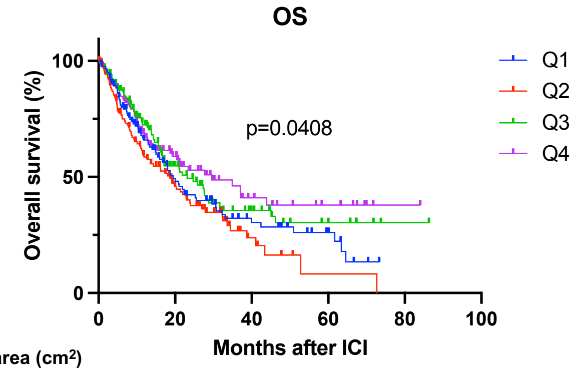

VAT area (cm^2)	No at risk					
(4.5 – 56.1)	125	42	16	8	0	0
(>56.1 – 112)	126	39	7	1	0	0
(>112 – 172)	124	42	17	8	1	0
(>172 – 470)	125	44	14	7	1	0

VAT area (cm^2)	Median OS in months (95% CI)	HR (95%CI)
Q1 (4.5 – 56.1)	19.5 (15.0, 25.5)	
Q2 (>56.1 – 112)	18.6 (11.5 - 22.9)	1.19 (0.87 - 1.65), p=0.28
Q3 (>112 –172)	23.1 (16.4 - 28.8)	0.83 (0.59 – 1.16), p=0.28
Q4 (>172 – 470)	29.6 (19.3 - NA)	0.76 (0.53 – 1.07), p=0.12

C. D.

EC patients treated with ICI stratified by SAT area (quartiles)

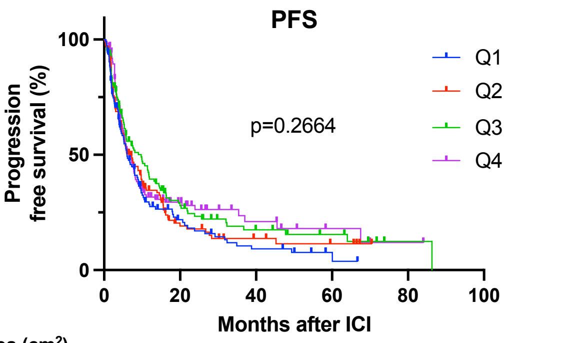

SAT area (cm^2)	No at risk					
(3 – 189)	125	18	7	2	0	0
(>189 – 270)	126	15	7	4	0	0
(>270 – 380)	124	25	10	6	1	0
(>380 – 866)	125	21	8	3	1	0

SAT area (cm^2)	Median PFS in months (95% CI)	HR (95%CI)
Q1 (3 – 189)	5.8 (4.2 - 8.0)	
Q2 (>189 – 270)	5.8 (4.4 - 8.9)	0.91 (0.69 - 1.21), p=0.53
Q3 (>270 –380)	7.8 (5.3 - 11.6)	0.78 (0.59 – 1.04), p=0.09
Q4 (>380 – 866)	6.4 (4.9 - 7.9)	0.79 (0.60 – 1.06), p=0.12

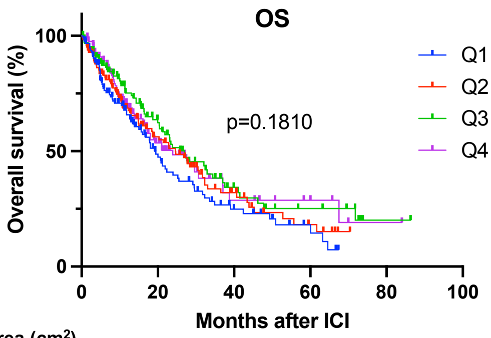

SAT area (cm^2)	No at risk					
(3 – 189)	125	38	13	5	0	0
(>189 – 270)	126	45	17	6	0	0
(>270 – 380)	124	48	15	8	1	0
(>380 – 866)	125	36	9	5	1	0

SAT area (cm^2)	Median OS in months (95% CI)	HR (95%CI)
Q1 (3 – 189)	18.0 (13.5 - 21.2)	
Q2 (>189 – 270)	22.9 (14.6 - 30.8)	0.85 (0.62 - 1.18), p=0.34
Q3 (>270 –380)	23.4 (16.7 - 41.1)	0.71 (0.50 – 0.99), p=0.04
Q4 (>380 – 866)	21.1 (16.2 - 37.0)	0.75 (0.53 – 1.06), p=0.10

Supplemental Figure 2. Survival outcomes after ICI in EC stratified by VAT and SAT area quartiles. Kaplan-Meier curves for (A) PFS and (B) OS in patients with EC following ICI treatment stratified by VAT area quartiles (Q1, 4.5 – 56.1 cm^2 in blue; Q2, >56.1 – 112 cm^2 in red; Q3, >112 – 172 cm^2 in green; Q4, >172 – 470 cm^2 in purple) (n=500). Kaplan-Meier curves for (C) PFS and (D) OS in patients with EC following ICI treatment stratified by SAT area quartiles SAT area quartiles (Q1, 3 – 189 cm^2 in blue; Q2, >189 – 270 cm^2 in red; Q3, >270 – 380 cm^2 in green; Q4, >380 – 866 cm^2 in purple) (n=500). The P values in the PFS and OS plots were calculated using a log-rank test. HRs and 95% CIs were calculated using Q1 as a reference. BMI, body mass index; OS, overall survival; PFS, progression free survival; EC, endometrial cancer; ICI, immune checkpoint inhibitor; VAT, visceral adipose tissue; SAT, subcutaneous adipose tissue; HR, Hazard ratio; CI, confidence interval.

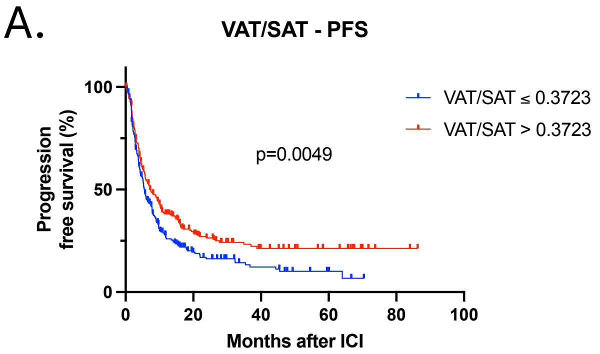

No at risk

VAT/SAT						
≤0.3723	250	30	12	4	0	0
>0.3723	250	49	20	11	2	0

VAT/SAT	Median PFS in months (95% CI)	HR (95%CI)
≤0.3723	5.5 (4.8 – 6.7)	
>0.3723	7.25 (5.8 – 9.9)	0.75 (0.61 – 0.92)

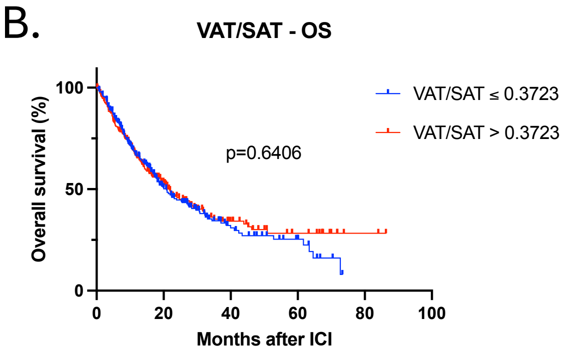

No at risk

VAT/SAT						
≤0.3723	250	85	25	11	0	0
>0.3723	250	82	29	13	2	0

VAT/SAT	Median OS in months (95% CI)	HR (95%CI)
≤0.3723	20.86 (17.2 – 27)	
>0.3723	21.86 (16.4 – 27.7)	0.94 (0.74 – 1.2)

Supplemental Figure 3. Survival outcomes after ICI in EC stratified by VAT/SAT ratio. Kaplan-Meier curves for (A) PFS and (B) OS in patients with EC following ICI treatment stratified by VAT/SAT ratio (n=500) (Low VAT/SAT ratio: ≤0.3723 in blue; High VAT/SAT ratio: >0.3723 in red). Patients were categorized as low or high VAT /SAT based on the median SAT and VAT of the entire cohort. The P values in the in PFS and OS plots were calculated using a log-rank test. BMI, body mass index; OS, overall survival; PFS, progression free survival; EC, endometrial cancer; ICI, immune checkpoint inhibitor; VAT, visceral adipose tissue; SAT, subcutaneous adipose tissue; HR, Hazard ratio; CI, confidence interval.

A.

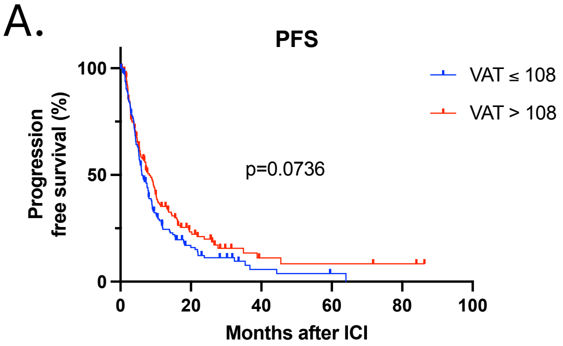

VAT area (cm²)

No at risk

≤108	149	17	3	1	0	0
>108	147	22	4	3	2	0

VAT area (cm²)	Median PFS in months (95% CI)	HR (95%CI)
≤108	6.14 (5.3 – 8)	
>108	8.21 (5.8 – 9.9)	0.79 (0.61 – 1.02)

B.

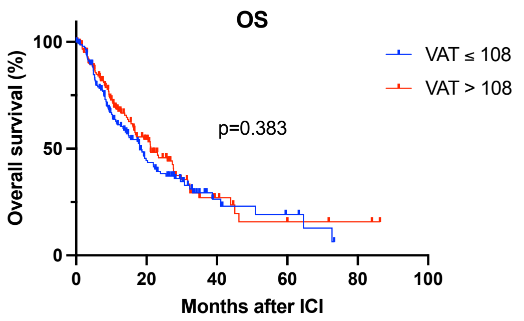

VAT area (cm²)

No at risk

≤108	149	44	8	4	0	0
>108	147	47	9	4	2	0

VAT area (cm²)	Median OS in months (95% CI)	HR (95%CI)
≤108	18.04 (13.5 – 22.4)	
>108	21.18 (16.4 – 27.7)	0.87 (0.64 – 1.19)

C.

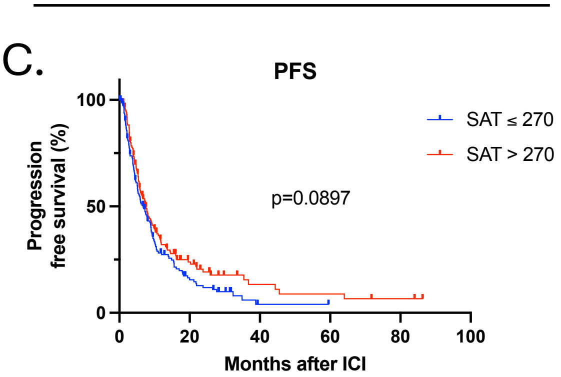

SAT area (cm²)

No at risk

≤270	150	17	1	0	0	0
>270	146	22	6	4	2	0

SAT area (cm²)	Median PFS in months (95% CI)	HR (95%CI)
≤270	6.75 (5.1 – 8.9)	
>270	7.5 (5.8 – 9.3)	0.8 (0.62 – 1.04)

D.

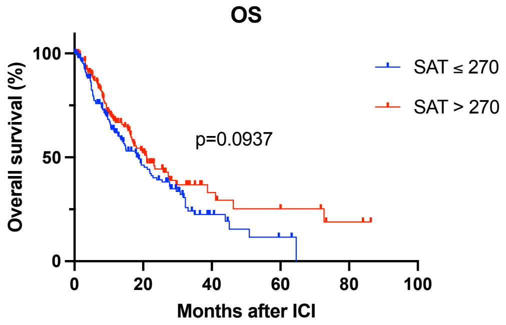

SAT area (cm²)

No at risk

≤270	150	46	8	2	0	0
>270	146	45	9	6	2	0

SAT area (cm²)	Median OS in months (95% CI)	HR (95%CI)
≤270	18.68 (13.5 – 22.9)	
>270	21 (16.7 – 27.6)	0.77 (0.56 – 1.05)

Supplemental Figure 4. Survival outcomes after Pembrolizumab and Lenvatinib in EC stratified by VAT and SAT area. Kaplan-Meier curves for (A) PFS and (B) OS in patients with EC following ICI treatment stratified by low and high VAT area (n=296) (Low VAT area: ≤108 cm² in blue; High VAT area: >108 cm² in red). Kaplan-Meier curves for (C) PFS and (D) OS in patients with EC following ICI treatment stratified by low and high SAT area (Low SAT area: ≤270 cm² in blue; High SAT area: >270 cm² in red) (n=500). Patients were categorized as low or high VAT /SAT based on the median SAT and VAT of the patient sub-group treated with Pembrolizumab + Lenvatinib. The P values in the PFS and OS plots were calculated using a log-rank test. BMI, body mass index; OS, overall survival; PFS, progression free survival; EC, endometrial cancer; ICI, immune checkpoint inhibitor; VAT, visceral adipose tissue; SAT, subcutaneous adipose tissue; HR, Hazard ratio; CI, confidence interval

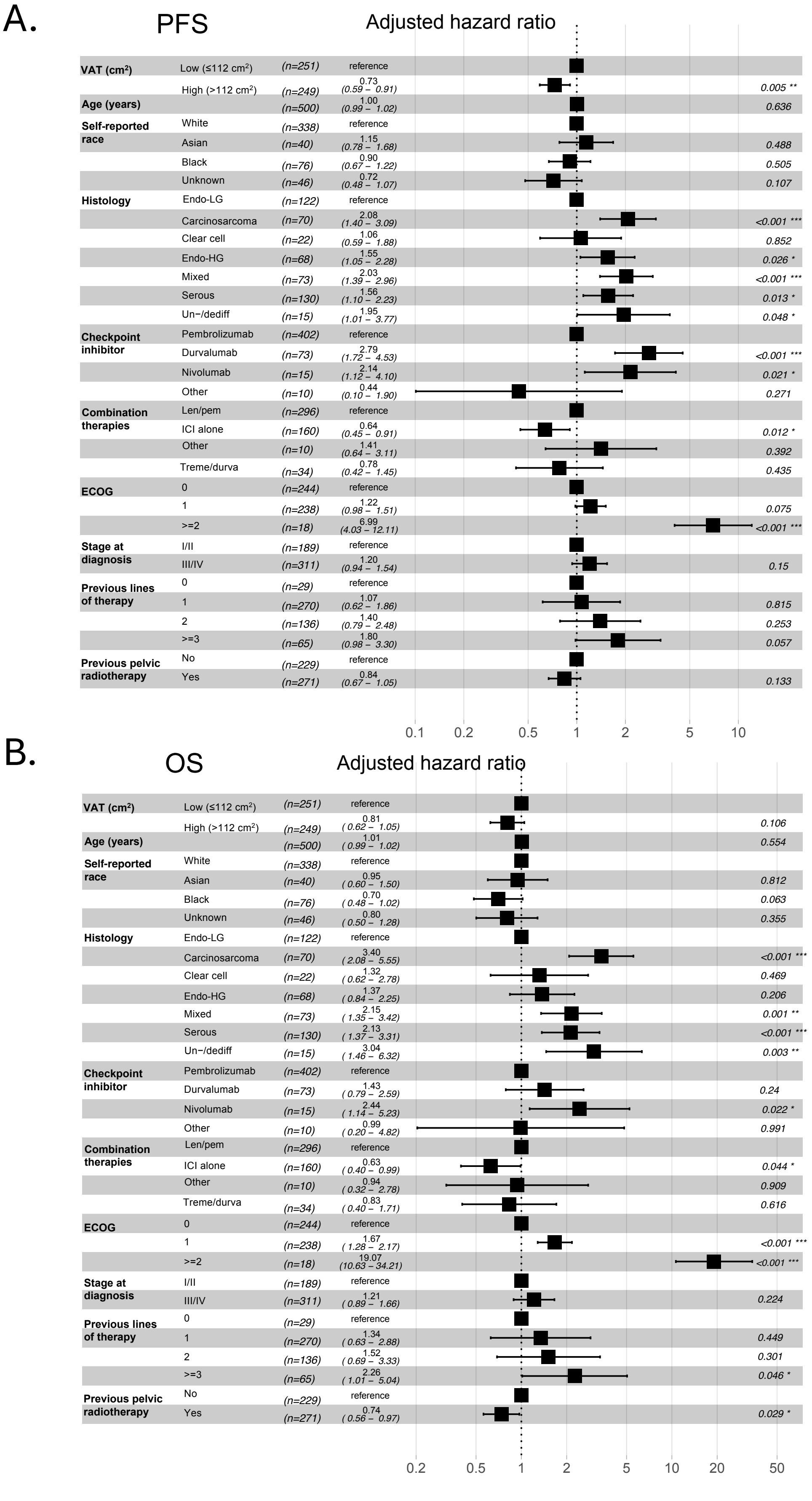

Supplemental Figure 5. Multivariable Cox regression analysis of VAT and other clinical variables associated with responses to ICI in EC patients. Forest plots of adjusted HRs and 95% CIs for patients with low VAT (reference group) compared to high VAT for (A) PFS and (B) OS (n=500). Analysis was adjusted for age, race, histology, type of checkpoint inhibitor, combination therapies, baseline performance status, stage at diagnosis, prior lines of therapy, and previous pelvic radiation. BMI, body mass index; OS, overall survival; PFS, progression free survival; EC, endometrial cancer; ICI, immune checkpoint inhibitor; VAT, visceral adipose tissue; Endo-LG, endometrial low grade; Endo-HG, endometrial high grade; Un-/dediff, Un-/dedifferentiated; Len/pem, Lenvatinib/pembrolizumab; Treme/durva, Tremelimumab/durvalumab; HR, Hazard ratio; CI, confidence interval.

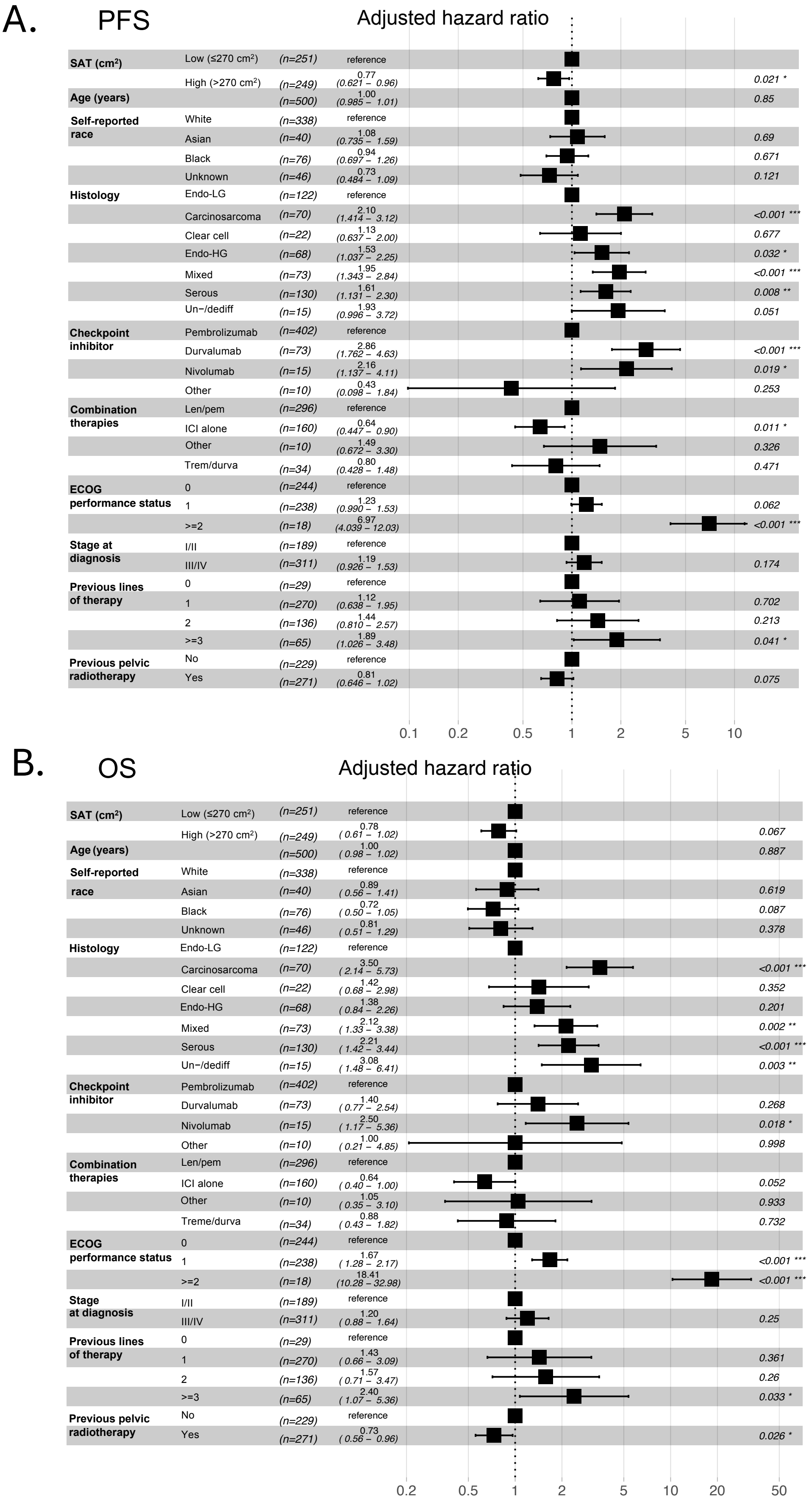

Supplemental Figure 6. Multivariable Cox regression analysis of SAT and other clinical variables associated with responses to ICI in EC patients. Forest plots of adjusted HRs and 95% Cis for patients with low SAT (reference group) compared to high SAT for (A) PFS and B) OS (n=500). Analysis was adjusted for age, self-reported race, histology, type of checkpoint inhibitor, combination therapies, baseline performance status, stage at diagnosis, prior lines of therapy, and previous pelvic radiation. BMI, body mass index; OS, overall survival; PFS, progression free survival; EC, endometrial cancer; ICI, immune checkpoint inhibitor; SAT, subcutaneous adipose tissue; Endo-LG, endometrial low grade; Endo-HG, endometrial high grade; Un-/dediff, Un-/dedifferentiated; Len/pem, Lenvatinib/pembrolizumab; Treme/durva, Tremelimumab/durvalumab; HR, Hazard ratio; CI, confidence interval.

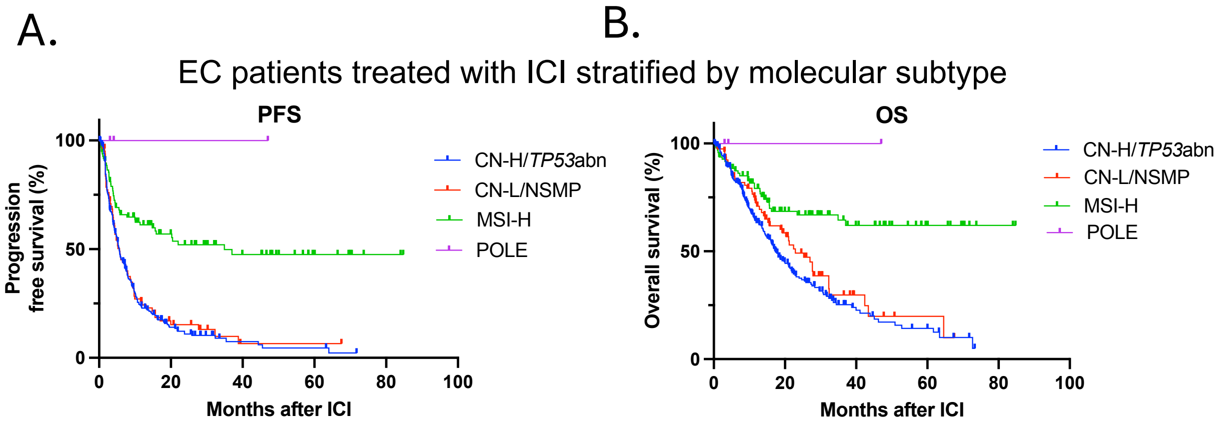

Supplemental Figure 7. Survival outcomes in patients with EC stratified by molecular classification after ICI treatment. Kaplan-Meier curves for (A) OS and (B) PFS in patients with EC following ICI treatment stratified by molecular subtype (n=437). OS, overall survival; PFS, progression free survival; EC, endometrial cancer; ICI, immune checkpoint inhibitor; CN-H/*TP53*abn, copy number-high/*TP53* abnormal; CN-L/NSMP, copy number-low/no specific molecular profile; MSI-H, microsatellite instability-high.

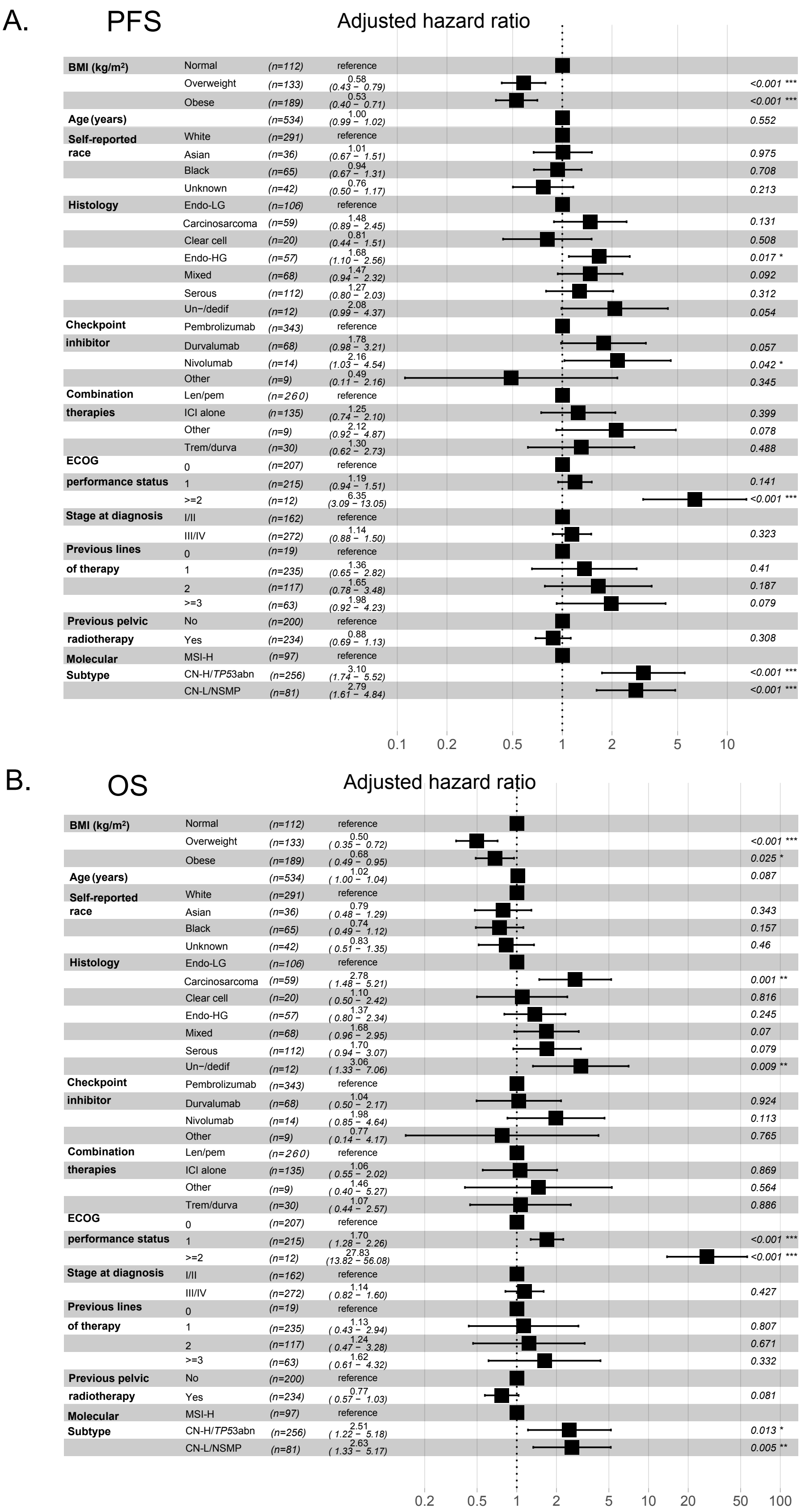

Supplemental Figure 8. Multivariable regression analysis of clinical and molecular signatures associated with responses to ICI in EC patients. Forest plots of adjusted HRs and 95% CIs for patients with normal BMI (18.5 – 24.9 kg/m^2) (reference) compared to overweight (BMI 25 – 29.9 kg/m^2) and obese (BMI > 30 kg/m^2) patients for (A) PFS and (B) (OS) (n=434). There were a limited number of POLE patients (n=3), so they were not included in the analysis. Analysis was adjusted for age, self-reported race, histology, type of checkpoint inhibitor, combination therapies, baseline performance status, stage at diagnosis, prior lines of therapy, previous pelvic radiation and molecular subtype. BMI, body mass index; OS, overall survival; PFS, progression free survival; EC, endometrial cancer; ICI, immune checkpoint inhibitor; Endo-LG, endometrial low grade; Endo-HG, endometrial high grade; Un-/dediff, Un-/dedifferentiated; Len/pem, Lenvatinib/pembrolizumab; Treme/durva, Tremelimumab/durvalumab; CN-H/*TP53*abn, copy number-high/*TP53* abnormal; CN-L/NSMP, copy number-low/no specific molecular profile; MSI-H, microsatellite instability-high; HR, Hazard ratio; CI, confidence interval.

A.

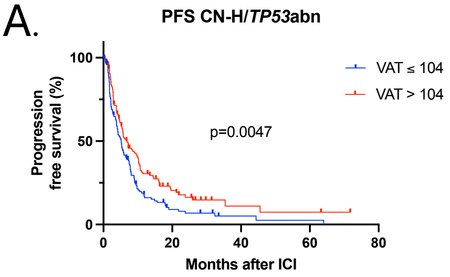

No at risk

VAT area (cm^2)					
≤104	125	8	2	1	0
>104	124	16	3	2	0

VAT area (cm^2)	Median PFS in months (95% CI)	HR (95%CI)
≤104	5.18 (3.9 – 6.5)	
>104	6.86 (5.1 – 9.8)	0.68 (0.51 – 0.89)

B.

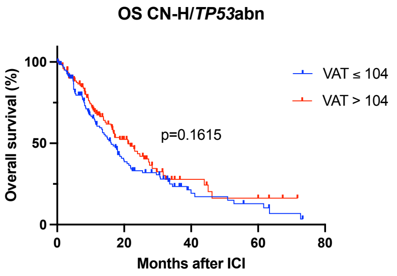

No at risk

VAT area (cm^2)					
≤104	125	36	9	5	0
>104	124	39	8	4	0

VAT area (cm^2)	Median OS in months (95% CI)	HR (95%CI)
≤104	16.14 (12.7 – 19.9)	
>104	21.18 (16.4 – 27.3)	0.79 (0.58 – 1.1)

C.

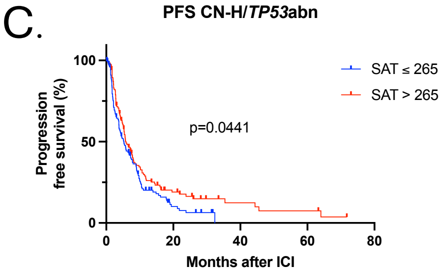

No at risk

SAT area (cm^2)					
≤265	125	8	0	0	0
>265	124	16	5	3	0

SAT area (cm^2)	Median PFS in months (95% CI)	HR (95%CI)
≤265	5.25 (3.8 – 7.3)	
>265	5.93 (5.1 – 7.8)	0.75 (0.57 – 0.99)

D.

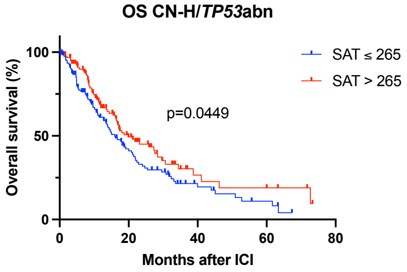

No at risk

SAT area (cm^2)					
≤265	125	37	10	4	0
>265	124	38	7	5	0

SAT area (cm^2)	Median OS in months (95% CI)	HR (95%CI)
≤265	15.96 (12.8 – 20.9)	
>265	19.86 (16.4 – 28.4)	0.72 (0.52 – 0.99)

Supplemental Figure 9. Survival outcomes in CN-H/*TP53*abnl EC stratified by VAT and SAT area. Kaplan-Meier curves for (A) PFS and (B) OS in patients with EC following ICI treatment stratified by low and high VAT area (n=249) (Low VAT area: ≤104 cm^2 in blue; High VAT area: >104 cm^2 in red). Kaplan-Meier curves for (C) PFS and (D) OS in patients with EC following ICI treatment stratified by low and high SAT area (Low SAT area: ≤265 cm^2 in blue; High SAT area: >265 cm^2 in red) (n=249). Patients were categorized as low or high VAT /SAT based on the median SAT and VAT area of the CN-H/*TP53*abn EC patients with available radiological data. The P values were calculated using a log-rank test. HRs and 95% CIs for overweight and obese patients were calculated using normal weight as a reference. BMI, body mass index; VAT, visceral adipose tissue; SAT, subcutaneous adipose tissue; OS, overall survival; PFS, progression free survival; EC, endometrial cancer; ICI, immune checkpoint inhibitor; CN-H/*TP53*abnl, copy number-high/*TP53*abnormal; HR, Hazard ratio; CI, confidence interval.

A.

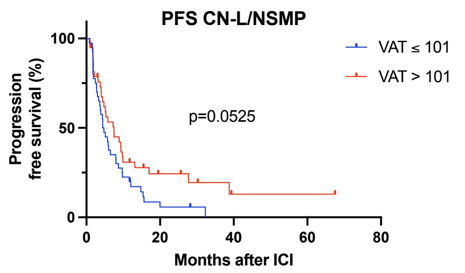

VAT area (cm^2)	No at risk				
≤101	40	2	0	0	0
>101	39	6	1	1	0

VAT area (cm^2)	Median PFS in months (95% CI)	HR (95%CI)
≤101	4.59 (3.3 – 6.5)	
>101	7.46 (4 – 9.8)	0.62 (0.38 – 1.01)

B.

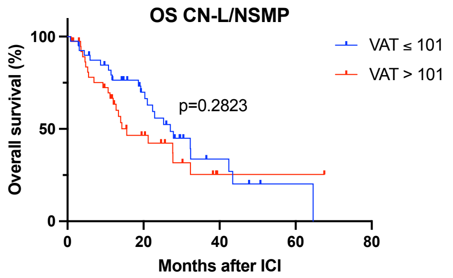

VAT area (cm^2)	No at risk				
≤101	40	20	5	1	0
>101	39	12	1	1	0

VAT area (cm^2)	Median OS in months (95% CI)	HR (95%CI)
≤101	27.04 (20.3 – 42.4)	
>101	15.57 (11.2 – 27.7)	1.38 (0.77 – 2.5)

C.

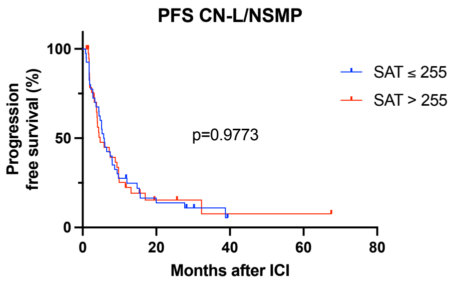

SAT area (cm^2)	No at risk				
≤255	40	5	0	0	0
>255	39	3	1	1	0

SAT area (cm^2)	Median PFS in months (95% CI)	HR (95%CI)
≤255	5.8 (4.4 – 8)	
>255	4.75 (3.8 – 9.3)	1.01 (0.62 – 1.63)

D.

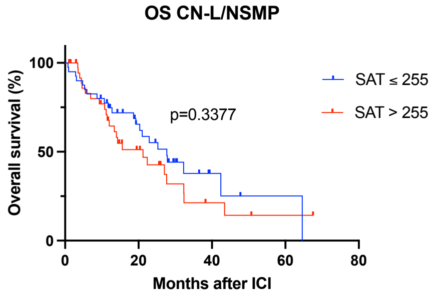

SAT area (cm^2)	No at risk				
≤255	40	19	3	1	0
>255	39	13	3	1	0

SAT area (cm^2)	Median OS in months (95% CI)	HR (95%CI)
≤255	27.71 (19.3– 42.4)	
>255	21.21 (11.5 – 32.3)	1.33 (0.74 – 2.4)

Supplemental Figure 10. Survival outcomes in CN-L/NSMP EC stratified by VAT and SAT area. Kaplan-Meier curves for (A) PFS and (B) OS in patients with EC following ICI treatment stratified by low and high VAT area (n=79) (Low VAT area: ≤101 cm^2 in blue; High VAT area: >101 cm^2 in red). Kaplan-Meier curves for (C) PFS and (D) OS in patients with EC following ICI treatment stratified by low and high SAT area (Low SAT area: ≤255 cm^2 in blue; High SAT area: >255 cm^2 in red) (n=79). Patients were categorized as low or high VAT /SAT based on the median SAT and VAT area of the CN-L/NSMP EC patients with available radiological data. The P values were calculated using a log-rank test. HRs and 95% CIs for overweight and obese patients were calculated using normal weight as a reference. BMI, body mass index; VAT, visceral adipose tissue; SAT, subcutaneous adipose tissue; OS, overall survival; PFS, progression free survival; EC, endometrial cancer; ICI, immune checkpoint inhibitor; CN-L/NSMP, copy number-low/no specific molecular profile; HR, Hazard ratio; CI, confidence interval.

A.

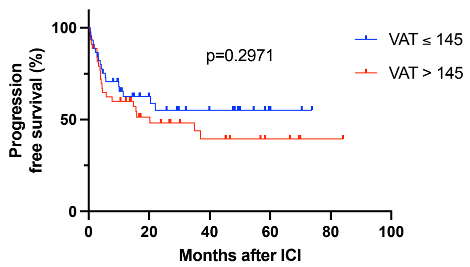

VAT area (cm²)	No at risk					
≤145	45	17	10	3	0	0
>145	45	16	9	4	1	0

VAT area (cm²)	Median OS in months (95% CI)	HR (95%CI)
≤145	Not reached	
>145	20.25 (4.6 - NA)	1.39 (0.75 – 2.57)

B.

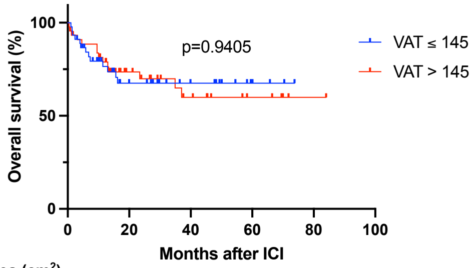

VAT area (cm²)	No at risk					
≤145	45	19	11	4	0	0
>145	45	21	11	5	1	0

VAT area (cm²)	Median OS in months (95% CI)	HR (95%CI)
≤145	Not reached	
>145	Not reached	1.03 (0.48 – 2.19)

C.

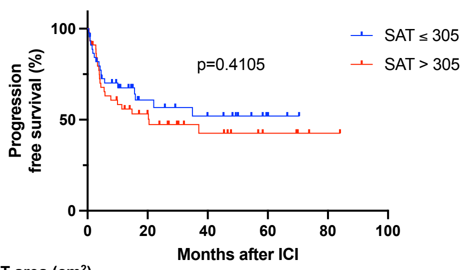

SAT area (cm²)	No at risk					
≤305	45	15	10	3	0	0
>305	45	18	9	4	1	0

SAT area (cm²)	Median PFS in months (95% CI)	HR (95%CI)
≤305	Not reached	
>305	20.46 (5.5 - NA)	1.29 (0.7 – 2.4)

D.

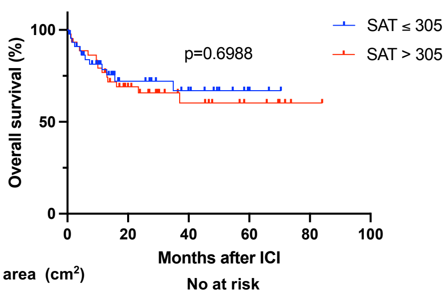

SAT area (cm²)	No at risk					
≤305	45	18	11	3	0	0
>305	45	22	11	6	1	0

SAT area (cm²)	Median OS in months (95% CI)	HR (95%CI)
≤305	Not reached	
>305	Not reached	1.16 (0.54 – 2.48)

Supplemental Figure 11. Survival outcomes in MSI-H EC stratified by VAT and SAT area. Kaplan-Meier curves for (A) PFS and (B) OS in patients with EC following ICI treatment stratified by low and high VAT area (n=90) (Low VAT area: ≤145 cm² in blue; High VAT area: >145 cm² in red). Kaplan-Meier curves for (C) PFS and (D) OS in patients with EC following ICI treatment stratified by low and high SAT area (Low SAT area: ≤305 cm² in blue; High SAT area: >305 cm² in red) (n=90). Patients were categorized as low or high VAT /SAT based on the median SAT and VAT area of the MSI-H EC patients with available radiological data. The P values were calculated using a log-rank test. HRs and 95% CIs for overweight and obese patients were calculated using normal weight as a reference. BMI, body mass index; VAT, visceral adipose tissue; SAT, subcutaneous adipose tissue; OS, overall survival; PFS, progression free survival; EC, endometrial cancer; ICI, immune checkpoint inhibitor; MSI-H, microsatellite instability-high; HR, Hazard ratio; CI, confidence interval.

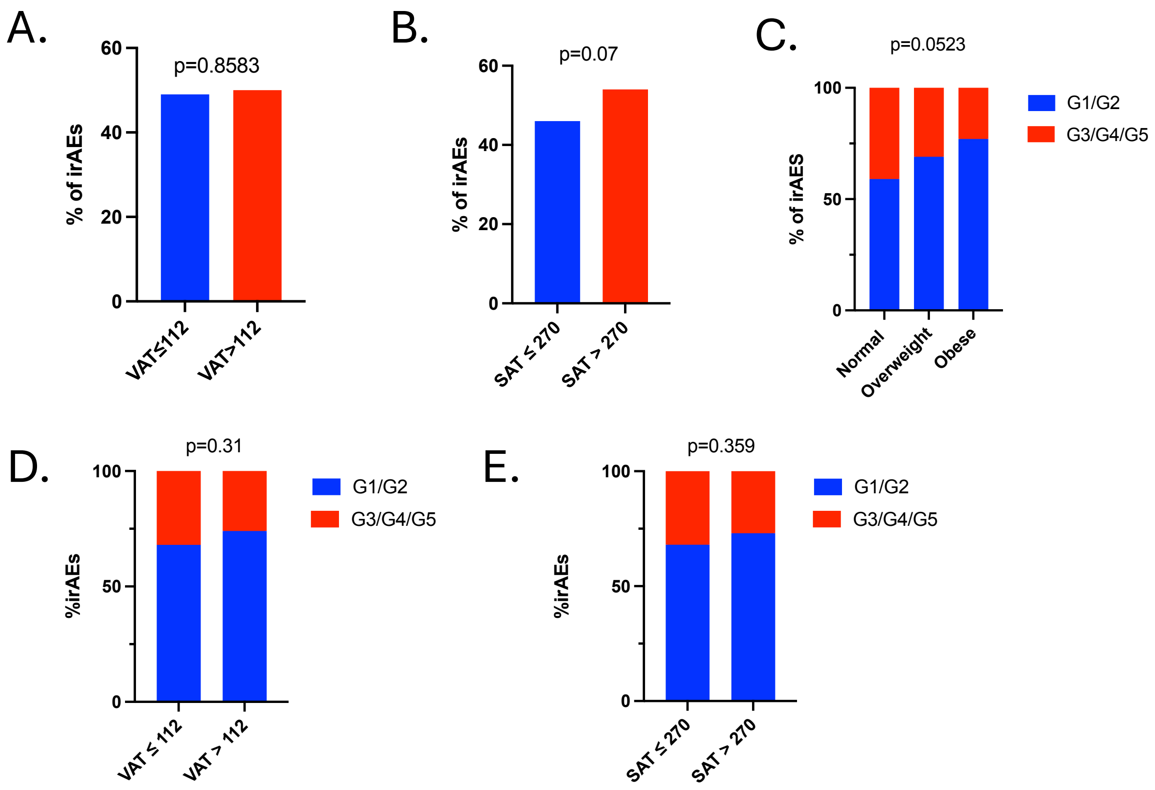

Supplemental Figure 12. Incidence of irAEs in EC patients after treatment with ICI stratified by BMI, VAT and SAT Percentage of irAEs stratified by (A) VAT area and (B) SAT area (n=500). Percentage of mild/moderate (G1/G2) irAEs vs severe (G3/G4/G5) irAEs stratified by (C) BMI (D) VAT area, and (E) SAT area (n=500). The P value in the bar graph was calculated using chi-squared test. BMI, body mass index; VAT, visceral adipose tissue; SAT, subcutaneous adipose tissue; EC, endometrial cancer; ICI, immune checkpoint inhibitor; iRAEs, immune related adverse events; G1/G2, Grade 1 and Grade 2; G3/G4/G5, Grade 3, Grade 4 and Grade 5.

	All n=437	Normal BMI n=114	Overweight n=133	Obese n=190	p value
**Median age (range)**	67 (40 – 94)	67 (41 – 89)	68 (43 – 94)	66 (40 – 88)	0.02
**Median BMI, kg/m^2 (range)**	28.8 (18.5 – 59.4)	22.5 (18.5 – 24.9)	27 (25 – 29.9)	35.1 (30 – 59.4)	< 0.0001
**Self-reported race - No (%)**<br>• White:<br>• Black:<br>• Asian:<br>• Unknown:	 293 (67)<br>65 (15)<br>36 (8)<br>43 (10)	 75 (66)<br>13 (11)<br>14 (12)<br>12 (11)	 99 (74)<br>17 (13)<br>12 (9)<br>5 (4)	 119 (63)<br>35 (18)<br>10 (5)<br>26 (14)	0.01
**Histology – No (%)**<br>• Endometroid<br>○ Low grade (1,2)<br>○ High grade (3)<br>• Serous<br>• Mixed/high-grade NOS<br>• Carcinosarcoma<br>• Clear Cell<br>• Un- / Dedifferentiated	 166 (38)<br>107 (24)<br>59 (14)<br>112 (26)<br>68 (16)<br>59 (14)<br>20 (5)<br>12 (3)	 38 (33)<br>27 (24)<br>11 (10)<br>23 (20)<br>17 (15)<br>22 (19)<br>7 (6)<br>7 (6)	 48 (36)<br>33 (25)<br>15 (11)<br>40 (30)<br>20 (15)<br>17 (13)<br>6 (5)<br>2 (2)	 80 (42)<br>47 (25)<br>33 (17)<br>49 (26)<br>31 (16)<br>20 (11)<br>7 (4)<br>3 (2)	0.12
**Checkpoint inhibitor – No (%)**<br>• Pembrolizumab:<br>• Durvalumab:<br>• Nivolumab:<br>• Other:	 346 (79)<br>68 (16)<br>14 (3)<br>9 (2)	 83 (73)<br>21 (18)<br>6 (5)<br>4 (4)	 106 (80)<br>22 (17)<br>4 (3)<br>1 (1)	 157 (83)<br>25 (13)<br>4 (2)<br>4 (2)	0.34
**Combination therapies – No (%)**<br>• Lenvatinib/pembrolizumab:<br>• Tremelimumab/durvalumab:<br>• ICI alone:<br>• Other combination:	 260 (59)<br>30 (7)<br>138 (32)<br>9 (2)	 62 (54)<br>10 (9)<br>38 (33)<br>4 (4)	 80 (60)<br>8 (6)<br>43 (32)<br>2 (2)	 118 (62)<br>12 (6)<br>57 (30)<br>3 (2)	0.76
**ECOG Performance Status– No (%)**<br>• 0:<br>• 1:<br>• 2-3	 208 (48)<br>217 (50)<br>12 (3)	 56 (49)<br>55 (48)<br>3 (3)	 73 (55)<br>56 (42)<br>4 (3)	 79 (42)<br>106 (56)<br>5 (3)	0.18
**Stage at diagnosis (1,2 vs 3,4) – No (%)**<br>• 1,2:<br>• 3,4:	 165 (38)<br>272 (62)	 41(36)<br>73 (64)	 50 (38)<br>83 (62)	 74 (39)<br>116 (61)	0.87
**Previous lines of therapy – No (%)**<br>• **0**<br>• **1**<br>• **2**<br>• **≥ 3**	 20 (5)<br>236 (54)<br>118 (27)<br>63 (14)	 8 (7)<br>50 (44)<br>35 (31)<br>21 (18)	 7 (5)<br>73 (55)<br>36 (27)<br>17 (13)	 5 (3)<br>113 (59)<br>47 (25)<br>25 (13)	0.17
**Previous pelvic radiotherapy – No (%)**<br>• Yes<br>• No	 237 (54)<br>200 (46)	 57 (50)<br>57 (50)	 80 (60)<br>53 (40)	 100 (53)<br>90 (47)	0.24
**Molecular subtype – No (%)**<br>• CN-H/*TP53*abn<br>• MSI-H<br>• CN-L/NSMP<br>• POLE	 256 (59)<br>97 (22)<br>81 (19)<br>3 (0.7)	 66 (58)<br>21 (18)<br>25 (22)<br>2 (2)	 77 (58)<br>25 (19)<br>31 (23)<br>0 (0)	 113 (59)<br>51 (27)<br>25 (13)<br>1 (0.5)	0.06

Suppplemental Table 1. Clinical characteristics of EC patients with available molecular subtype. BMI, body mass index; ECOG, Eastern Cooperative Oncology Group; CPN-H/*TP53*abn, copy number-high/*TP53*abnormal; CN-L/NSMP, copy number-low/no specific molecular profile; MSI-H, microsatellite instability high; VAT, visceral adipose tissue; SAT, subcutaneous adipose tissue. P values in table come from Kruskal-Wallis, chi-squared or Fisher's exact tests.

BMI (Kg/m^2)	Pneumonitis	Myocarditis	Neurological	Ocular	Hematologic
**Normal (18.5 – 24.9) – No (%)**	1 (0.8)	0 (0)	3 (2)	0 (0)	1 (0.8)
**Overweight (25 – 29.9) – No (%)**	1 (0.6)	2 (1)	0 (0)	1 (0.6)	1 (0.6)
**Obese (≥ 30) – No (%)**	2 (0.9)	1 (0.4)	2 (0.9)	1 (0.4)	0 (0)
**Total - No (%)**	4 (0.7)	3 (0.6)	5 (1)	2 (0.4)	2 (0.4)
**p value**	1	0.5	0.1	1	0.3

Supplemental Table 2. irAEs per organ system in EC patients after treatment with ICI stratified by BMI. Absolute number and percentage of irAEs per organ system across BMI categories Normal - BMI: 18.5 – 24.9 kg/m^2; Overweight – BMI 25 – 29.9 kg/m^2; Obese – BMI > 30 kg/m^2. P-values in were calculated with Fisher's exact test. BMI, body mass index; EC, endometrial cancer; ICI, immune checkpoint inhibitor; irAEs, immune related adverse events.
